# Senescence of endothelial cells increases susceptibility to Kaposi’s sarcoma–associated herpesvirus infection via CD109-mediated viral entry

**DOI:** 10.1172/JCI183561

**Published:** 2024-12-12

**Authors:** Myung-Ju Lee, Jun-Hee Yeon, Jisu Lee, Yun Hee Kang, Beom Seok Park, Joohee Park, Sung-Ho Yun, Dagmar Wirth, Seung-Min Yoo, Changhoon Park, Shou-Jiang Gao, Myung-Shin Lee

**Affiliations:** 1 Department of Microbiology and Immunology, and; 2 Eulji Biomedical Science Research Institute, Eulji University School of Medicine, Daejeon, South Korea.; 3 Department of Biomedical Laboratory Science, College of Health Science, Eulji University, Seongnam, South Korea.; 4 Research Center for Bioconvergence Analysis, Korea Basic Science Institute (KBSI), Cheongju, South Korea.; 5 Model Systems for Infection and Immunity, Helmholtz Centre for Infection Research, Braunschweig, Germany.; 6 Tumor Virology Program, UPMC Hillman Cancer Center, and Department of Microbiology and Molecular Genetics, University of Pittsburgh, Pittsburgh, Pennsylvania, USA.

**Keywords:** Aging, Virology, Cellular senescence, Endothelial cells, Molecular biology

## Abstract

The aging process is characterized by cellular functional decline and increased susceptibility to infections. Understanding the association between virus infection and aging is crucial for developing effective strategies against viral infections in older individuals. However, the relationship between Kaposi’s sarcoma–associated herpesvirus (KSHV) infection, a cause of increased Kaposi’s sarcoma prevalence among the elderly without HIV infection, and cellular senescence remains enigmatic. This study uncovered a link between cellular senescence and enhanced KSHV infectivity in human endothelial cells. Through a comprehensive proteomic analysis, we identified caveolin-1 and CD109 as host factors significantly upregulated in senescent cells that promote KSHV infection. Remarkably, CRISPR/Cas9-mediated KO of these factors reduced KSHV binding and entry, leading to decreased viral infectivity. Furthermore, surface plasmon resonance analysis and confocal microscopy revealed a direct interaction between KSHV virions and CD109 on the cell surface during entry, with recombinant CD109 protein exhibiting inhibitory activity of KSHV infection by blocking virion binding. These findings uncover a previously unrecognized role of cellular senescence in enhancing KSHV infection through upregulation of specific host factors and provide insights into the complex interplay between aging and viral pathogenesis.

## Abstract 2

Cellular senescence enhances KSHV infection of endothelial cells by increasing surface expression of CD109, which directly binds to KSHV and promotes viral entry.

## Graphical abstract

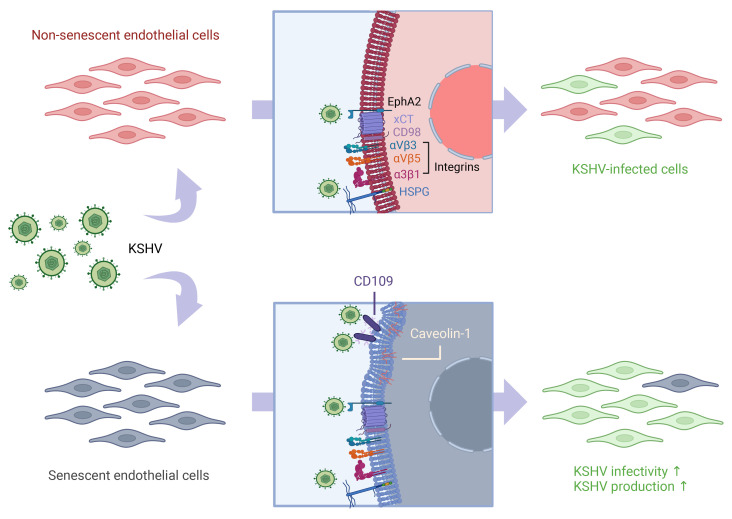

## Introduction

Kaposi’s sarcoma–associated herpesvirus (KSHV), also known as human herpesvirus 8 (HHV-8), is a member of the herpesvirus family. KSHV infection is associated with the development of Kaposi’s sarcoma (KS), a vascular cancer that is particularly prevalent in immunocompromised individuals, including those with AIDS (1). KS is characterized by the formation of red or purple skin lesions, which can progress to involve the internal organs and cause serious complications. Classic KS is more common in older individuals, particularly those over the age of 50 years (2). The incidence of KS increases with age, and the disease is more aggressive and difficult to treat in older patients (3). This age-dependent pattern suggests a complex interplay between viral pathogenesis and the aging process, the mechanisms of which remain elusive.

HIV infection is one of the most important risk factors for KS (4). HIV infection has been associated with an increased risk of age-related diseases, including cardiovascular disease, neurocognitive impairment, and certain cancers (5, 6). Cellular senescence, a hallmark of aging, has been proposed as a potential factor in the pathogenesis of various age-related diseases (7, 8). Some studies have suggested that HIV infection may influence various processes associated with cellular senescence (5, 9). HIV-induced oxidative stress and the direct effects of viral proteins, such as Tat, may contribute to premature cellular senescence (10). While the relationships among aging, HIV infection, and KS are complex and multifaceted, these findings highlight the potential impact of cellular senescence on KS. However, the specific role of senescence in KSHV infection and KS development is yet to be established. Given the age-dependent pattern of KS incidence and severity, understanding the relationship between cellular senescence and KSHV susceptibility could provide valuable insights into KS pathogenesis.

To address this knowledge gap, we investigated the susceptibility of senescent human endothelial cells to KSHV infection using primary HUVECs and a conditionally immortalized human endothelial cell line containing doxycycline-controlled SV40 large T antigen and hTERT-expressing cassettes (HuARLT cells) (11). We found that senescent cells are more susceptible to KSHV infection than nonsenescent cells. Mass spectrometry analysis revealed differential expression of proteins between senescent and nonsenescent cells, of which 4 membrane proteins were examined for their roles in the increased susceptibility of senescent cells to KSHV. Throughout systematic examination involving the use of KO clones and cells overexpressing candidate proteins, we discovered that CD109 had an essential role in KSHV attachment and infection in human endothelial cells, particularly in senescent cells.

## Results

### Cellular senescence enhances KSHV infectivity in human endothelial cells.

**Figure 1:**
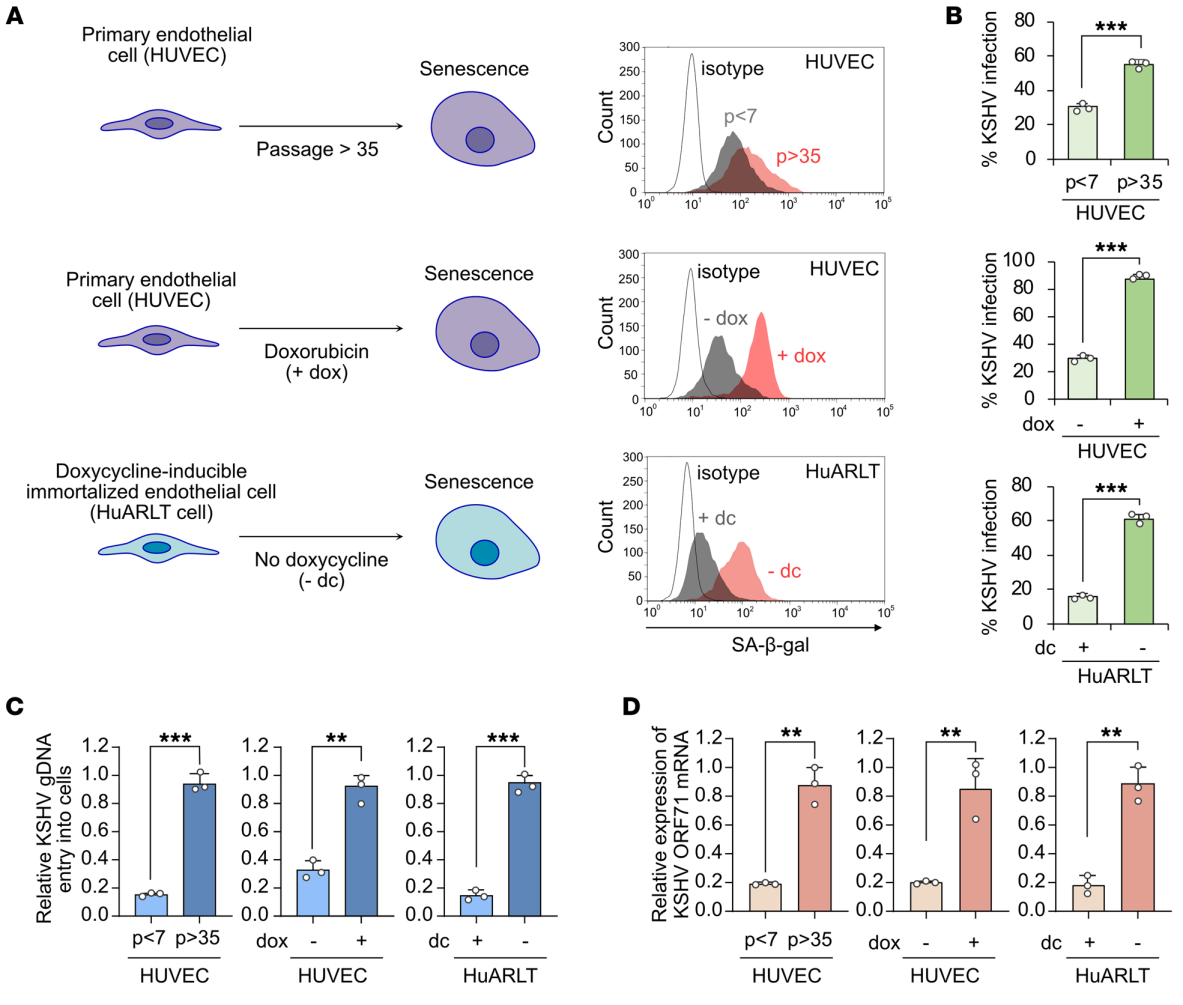
Increased KSHV infection in senescent human endothelial cells. (**A**) Senescence was induced in primary human endothelial cells (HUVECs) by repeated subculture over 35 passages or treatment with doxorubicin. In doxycycline-inducible immortalized human endothelial cells (HuARLT cells), senescence was induced by culturing without doxycycline. SA-β-gal staining was used for validation of senescence through microscopy or flow cytometry. p, cell culture passages. dc, doxycycline. KSHV infectivity was measured by GFP expression in cells infected with recombinant KSHV BAC16. (**B**) Analysis of KSHV-infected cells in nonsenescent and senescent human endothelial cells by flow cytometry at 24 hpi. Data are representative of 3 independent experiments. Data are shown as mean ± SD. *n* = 3. ^***^P < 0.001, unpaired 2-tailed Student’s *t* test. (**C**) Quantification of the KSHV genome in KSHV-infected nonsenescent and senescent human endothelial cells by quantitative PCR at 24 hpi. Data are representative of 3 independent experiments. Data are shown as mean ± SD. *n* = 3. ^**^P < 0.01, ^***^P < 0.001, unpaired 2-tailed Student’s *t* test. (**D**) Assessment of the relative expression of KSHV *ORF71* mRNA in KSHV-infected nonsenescent and senescent human endothelial cells using quantitative reverse transcription PCR at 24 hpi. Data are representative of 3 independent experiments. Data are shown as mean ± SD. *n* = 3. ^**^P < 0.01, unpaired 2-tailed Student’s *t* test.

To investigate the infectivity of KSHV in senescent human endothelial cells, we employed 3 established senescence models: replicative senescence in HUVECs through extended passaging (>35 passages), doxorubicin-induced senescence in HUVECs (12, 13), and doxycycline withdrawal–induced senescence in HuARLT cells (11) (Figure 1A). The senescent status of HUVECs and HuARLT cells was analyzed by SA-β-gal, Western blotting, and cell proliferation. Cultures induced for senescence by all 3 methods had increased SA-β-gal–positive cells (Figure 1A and Supplemental Figure 1A) and increased expression levels of p16 or p21 proteins (Supplemental Figure 1B). After 35 passages, senescent HUVECs had a lower rate of cell division compared with the early passage cells (Supplemental Figure 1C). Similarly, senescent cells derived from both doxycycline-withdrawn HuARLT cells and doxorubicin-treated HUVECs exhibited diminished cell proliferation compared with their nonsenescent control cells (Supplemental Figure 1D). In cell-cycle analysis, all senescent cells demonstrated decreased S phase cells (Supplemental Figure 1E). Together, these results confirm that all 3 approaches effectively triggered cellular senescence in human endothelial cells.

To assess the ability of senescent cells to support KSHV infection, the 3 types of senescent cells were infected with recombinant KSHV BAC16. Since senescent cells stop dividing, equal numbers of senescent and nonsenescent cells were seeded 1 day prior to infection to minimize differences in cell number. The next day, equal amounts of virus were used to infect cells, aiming to produce 10%–30% of GFP-positive cells in nonsenescent cells. Because recombinant KSHV BAC16 contains a GFP cassette, we monitored KSHV infection based on the expression of GFP protein at day 1 after infection (14). All 3 types of senescent endothelial cells showed significantly higher levels of KSHV infection than their nonsenescent control cells (Figure 1B and Supplemental Figure 2). These results were confirmed by staining for the expression of KSHV latent protein latent nuclear antigen (LANA) in the infected cells (Supplemental Figure 3). In agreement with these results, higher copy numbers of viral genomes and higher expression levels of viral latent gene *ORF71* were detected in KSHV-infected senescent than nonsenescent cells (Figure 1, C and D). We investigated whether senescent cells experienced a different outcome after infection with KSHV by examining their viability and the expression of viral lytic cycle genes at day 4 after infection. Intriguingly, after KSHV infection, the number of dead cells was significantly increased in senescent HUVECs and HuARLT cells compared with nonsenescent cells (Supplemental Figure 4, A and B). In senescent cells, mRNA and protein expressions of lytic genes of KSHV were significantly increased (Supplemental Figure 4, C and D). Furthermore, we found that virus production was also increased in senescent cells (Supplemental Figure 4E). These findings indicate that senescent cells are more susceptible to KSHV infection and support a higher level of viral lytic replication. Because of the challenges in maintaining a large quantity of HUVECs in the replicative senescence state, we primarily performed our subsequent experiments with doxorubicin-treated HUVECs and doxycycline-withdrawn HuARLT cells.

### Increased KSHV entry and binding in senescent endothelial cells.

**Figure 2:**
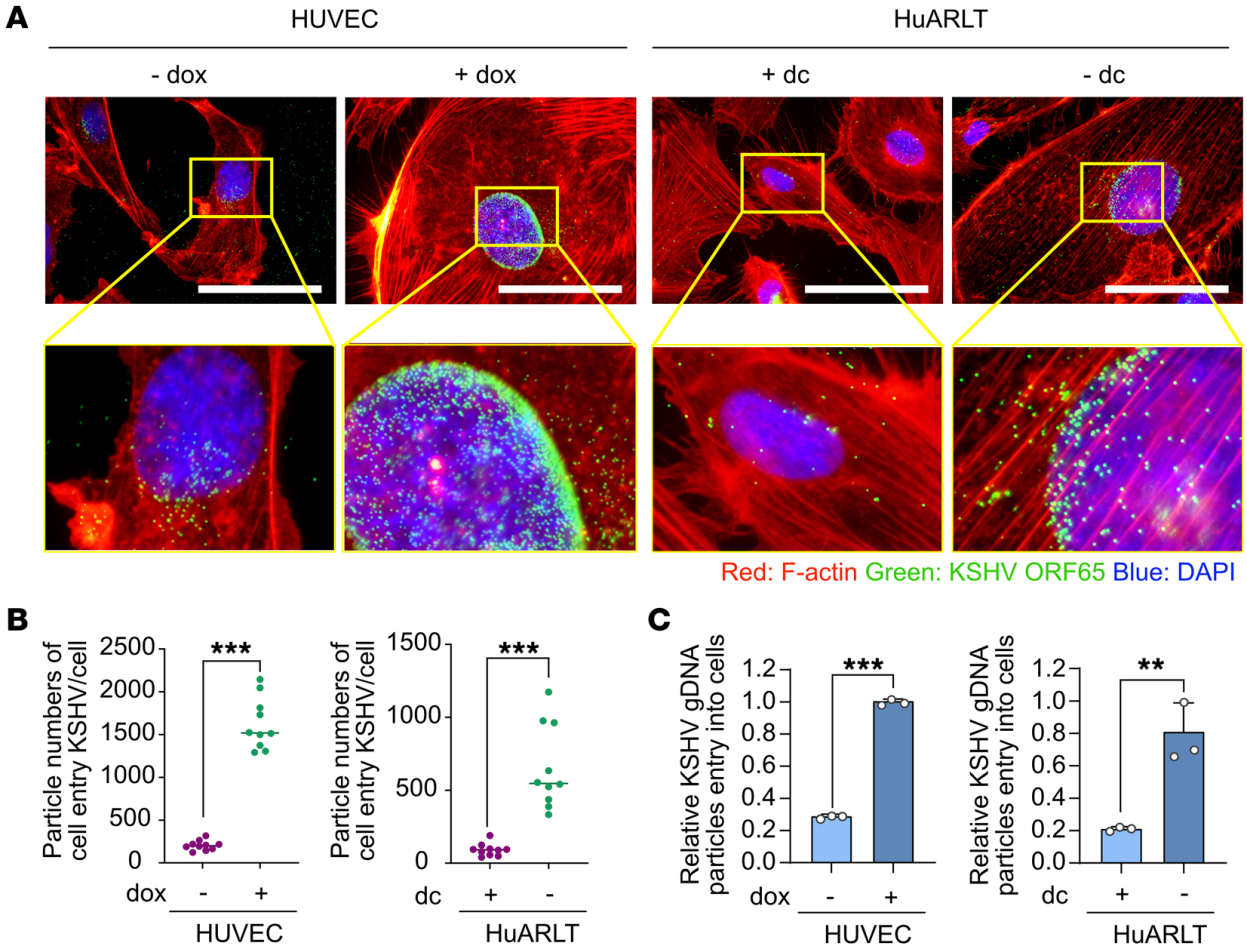
Enhanced entry of KSHV in senescent human endothelial cells. (**A**) Immunofluorescence assay of the entry of KSHV into nonsenescent and senescent cells. KSHV was visualized at 4 hpi using KSHV ORF65 antibody. Phalloidin (F-actin) and DAPI were used to visualize the shapes of the cells and nuclei, respectively. Scale bars: 50 μm. (**B**) Analysis of the number of internalized KSHV particles per cell in the images in **A** and Supplemental Figure 5. Data are representative of 10 independent experiments. Data are shown as mean ± SD. *n* = 10. ^***^P < 0.001 using unpaired 2-tailed Student’s *t* test. (**C**) Quantification of the internalized KSHV genome in the KSHV-infected nonsenescent and senescent human endothelial cells by quantitative PCR. Genomic DNA was extracted from KSHV-infected cells at 4 hpi. The KSHV genome was quantified in the extracted DNA by quantitative PCR using primers for KSHV ORF26. Data are representative of 3 independent experiments. Data are shown as mean ± SD. *n* = 3. ^**^P < 0.01; ^***^P < 0.001, unpaired 2-tailed Student’s *t* test.

To determine whether senescence might affect KSHV entry and trafficking, we examined KSHV particles by staining the viral capsid protein ORF65 at 4 hours postinfection (hpi) (Figure 2, A and B, and Supplemental Figure 5). A previous study has shown that KSHV particles dock at perinuclear regions following successful entry into and trafficking in cells (15). We found significantly more KSHV particles in perinuclear regions in senescent HUVECs and HuARLT cells than in nonsenescent control cells. Additionally, we detected higher copy numbers of KSHV genomic DNA in senescent than in nonsenescent cells (Figure 2C). These results indicate that senescence might facilitate KSHV entry and trafficking.

**Figure 3:**
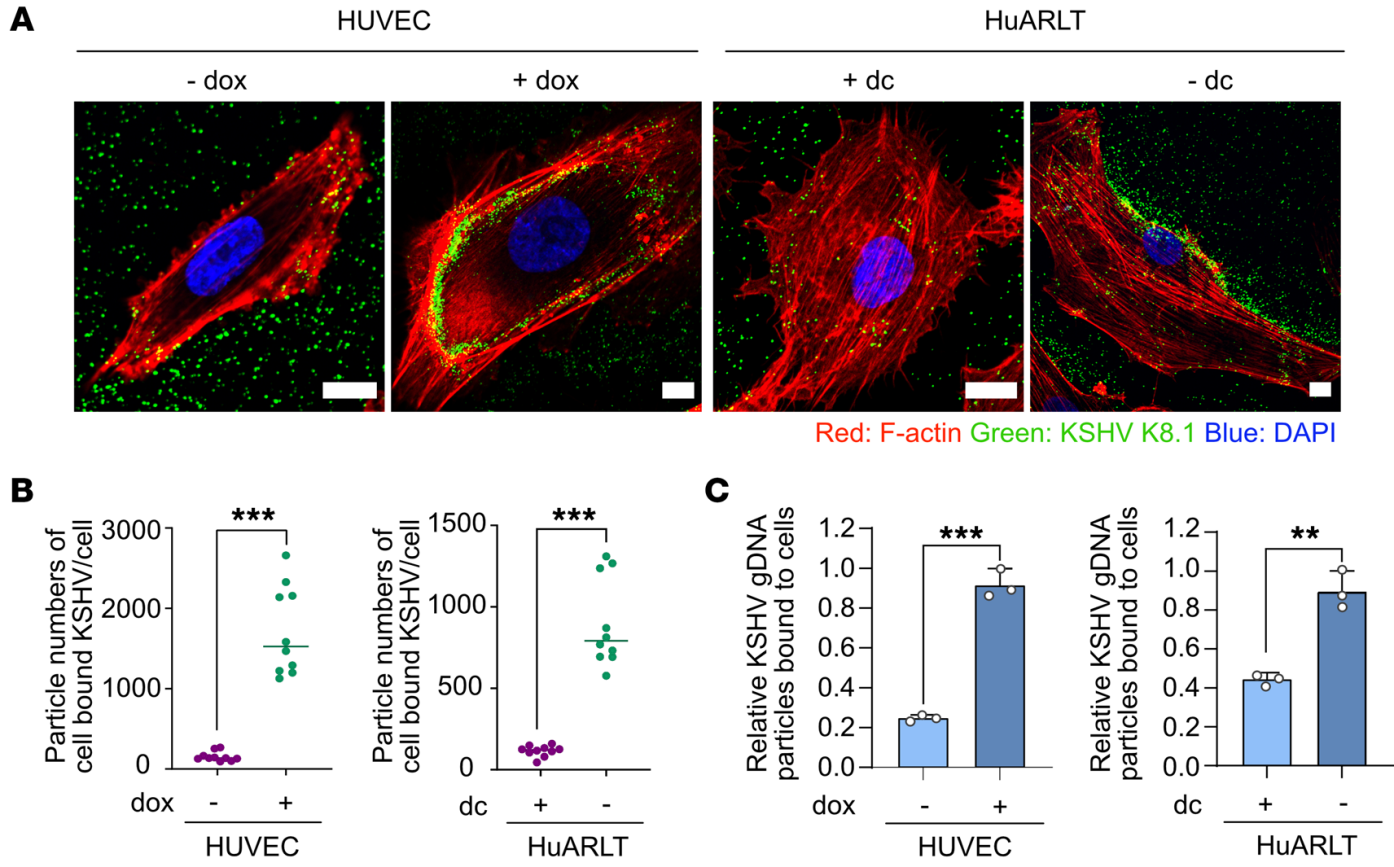
Enhanced binding of KSHV in senescent human endothelial cells. (**A**) Confocal microscopy images of KSHV binding to the cell surface. After infection, cells were immediately fixed and stained without permeabilization. Scale bars: 10 μm. (**B**) Analysis of the number of KSHV particles binding to the cell surface per cell in the images in **A** and Supplemental Figure 6. Data are representative of 10 independent experiments. Data are shown as mean ± SD. *n* = 10. ^***^P < 0.001, unpaired 2-tailed Student’s *t* test. (**C**) Quantification of the cell-surface–bound KSHV genome in KSHV-infected cells by quantitative PCR. After 1 hour of KSHV infection, the cells were immediately scraped and genomic DNA was extracted. The KSHV genome was quantified in the extracted DNA by quantitative PCR using primers for KSHV ORF26. Data are representative of 3 independent experiments. Data are shown as mean ± SD. *n* = 3. ^**^P < 0.01; ^***^P < 0.001, unpaired 2-tailed Student’s *t* test.

To investigate whether the enhanced KSHV entry and trafficking in senescent cells was due to increased binding of virions on the cell surface, we infected the cells with KSHV virions for 1 hour at 4°C. We then examined the virions by staining for KSHV envelope glycoprotein K8.1 without a permeabilization process (Figure 3, A and B, and Supplemental Figure 6). We found that KSHV binding to the cell surface was significantly increased in senescent cells compared with nonsenescent cells. Thus, the increased KSHV infectivity in senescent cells might be in part due to the enhanced binding of virions to the cell surface. To confirm these results, we isolated KSHV-infected cells at 1 hpi through a scraping and washing procedure. DNA was then extracted and quantified for viral genome copy numbers (Figure 3C). We detected higher viral genomic copy numbers in senescent than nonsenescent cells. Thus, senescence might promote KSHV infection by enhancing binding of virions to the cells.

KSHV enters host cells through a multistep process that involves interactions between viral envelope glycoproteins and host cell-surface receptors. Several endothelial cell-surface proteins have been identified as potential entry receptors for KSHV, including integrins, heparan sulfate proteoglycan (HSPG), and ephrin type-A (EphA) receptors (16). To investigate whether the expression of known receptors for KSHV entry was changed in senescent cells, we analyzed their expression levels by flow cytometry (Supplemental Figure 7). In HUVECs, the expression levels of numerous known KSHV entry receptors, such as EphA2 and integrin α_V_β_5_, were increased in senescent cells compared with nonsenescent cells. However, no known KSHV entry receptors were increased in senescent HuARLT cells. In contrast, numerous KSHV entry receptors, such as HSPG, xCT, and CD98, had reduced surface expression in senescent HuARLT cells compared with nonsenescent cells. Given these observations, there may be additional host factors responsible for the elevated susceptibility to KSHV infection in senescent endothelial cells.

### Identification of membrane proteins associated with increased KSHV infectivity in senescent cells.

**Figure 4:**
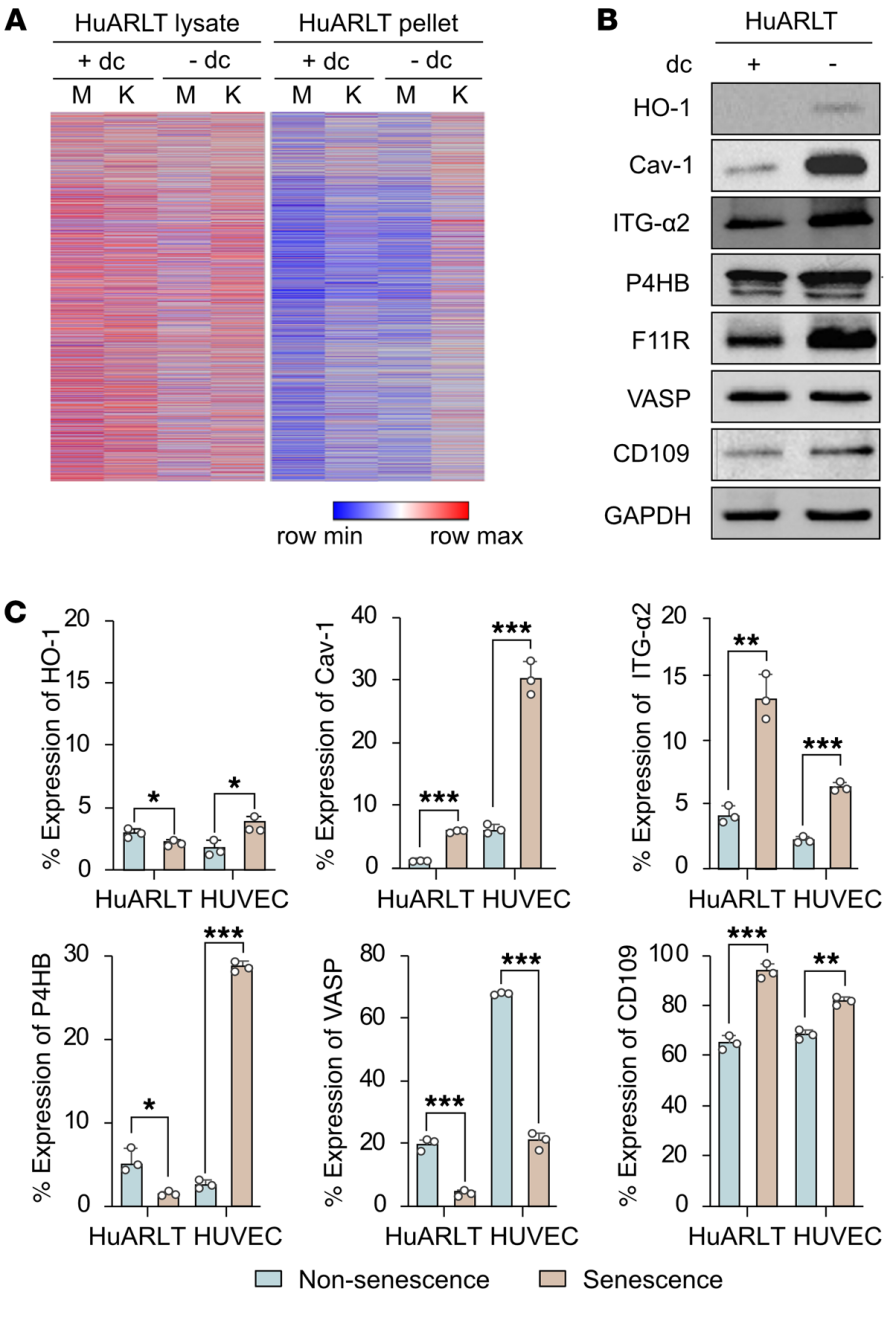
Identification of candidate proteins associated with increased KSHV infectivity in senescent endothelial cells. (**A**) Heatmap of the differential expression of proteins from nonsenescent (+dc, culture with doxycycline) and senescent (–dc, culture without doxycycline) HuARLT cells with KSHV (K) or without KSHV (M). The cell lysate and cell pellet from each conditioned cell were analyzed by LC-MS/MS. (**B**) Western blot analysis of the selected candidate proteins. Cav-1, caveolin-1; ITG, integrin; dc, doxycycline. GAPDH was used as a housekeeping protein for normalization. (**C**) Flow cytometric analysis of candidate proteins in nonsenescent (+dc HuARLT and –dox HUVEC) and senescent (–dc HuARLT and +dox HUVEC) cells. Data are representative of 3 independent experiments. Data are shown as mean ± SD. *n* = 3. ^*^P < 0.05; ^**^P < 0.01; ^***^P < 0.001, unpaired 2-tailed Student’s *t* test.

To identify host factors that might enhance KSHV infectivity in senescent human endothelial cells, we compared the differential protein expression between nonsenescent and senescent HuARLT cells by SDS-PAGE (Supplemental Figure 8). Because of the enhanced binding of KSHV in senescent cells (Figure 3, A–C), we analyzed both the soluble (cell lysate) and insoluble (cell pellet) fractions after protein lysis to ensure the inclusion of all membrane proteins. After Coomassie blue staining of the SDS-PAGE gel, we observed variations in protein expression between uninfected senescent and nonsenescent cells. Liquid chromatography/tandem mass spectrometry (LC-MS/MS) revealed differentially expressed proteins between senescent and nonsenescent cells (Figure 4A, Table 1, and Supplemental Table 1). A total of 6,575 proteins were identified in LC-MS/MS analysis. Of these, 303 proteins were increased more than 2-fold in the lysate of senescent cells compared with control cells. In addition, 45 proteins were increased more than 2-fold in the pellet from senescent cells compared with control cells. Following a thorough analysis of the LC-MS/MS data, 7 membrane proteins were chosen as potential host factors that could enhance KSHV infection in senescent endothelial cells (Table 1). To confirm the differential expression of the candidate proteins in control and senescent cells, they were analyzed by Western blotting (Figure 4B). Higher expression levels of all candidate proteins, except vasodilator stimulated phosphoprotein (VASP), were observed in senescent HuARLT cells compared with control cells. Cell-surface expression was analyzed in HuARLT cells and HUVECs using flow cytometry (Figure 4C and Supplemental Figure 9). The proteins that were increased in both senescent HuARLT cells and doxorubicin-treated HUVECs compared with their control cells included caveolin-1, integrin α_2_, F11R, and CD109. The expression rate of F11R was more than 100% in both nonsenescent and senescent cells compared with the isotype controls in flow cytometry, but senescent cells had higher expression levels (Supplemental Figure 9).

### Establishment and characterization of KO clones for the candidate proteins in HuARLT cells.

**Figure 5:**
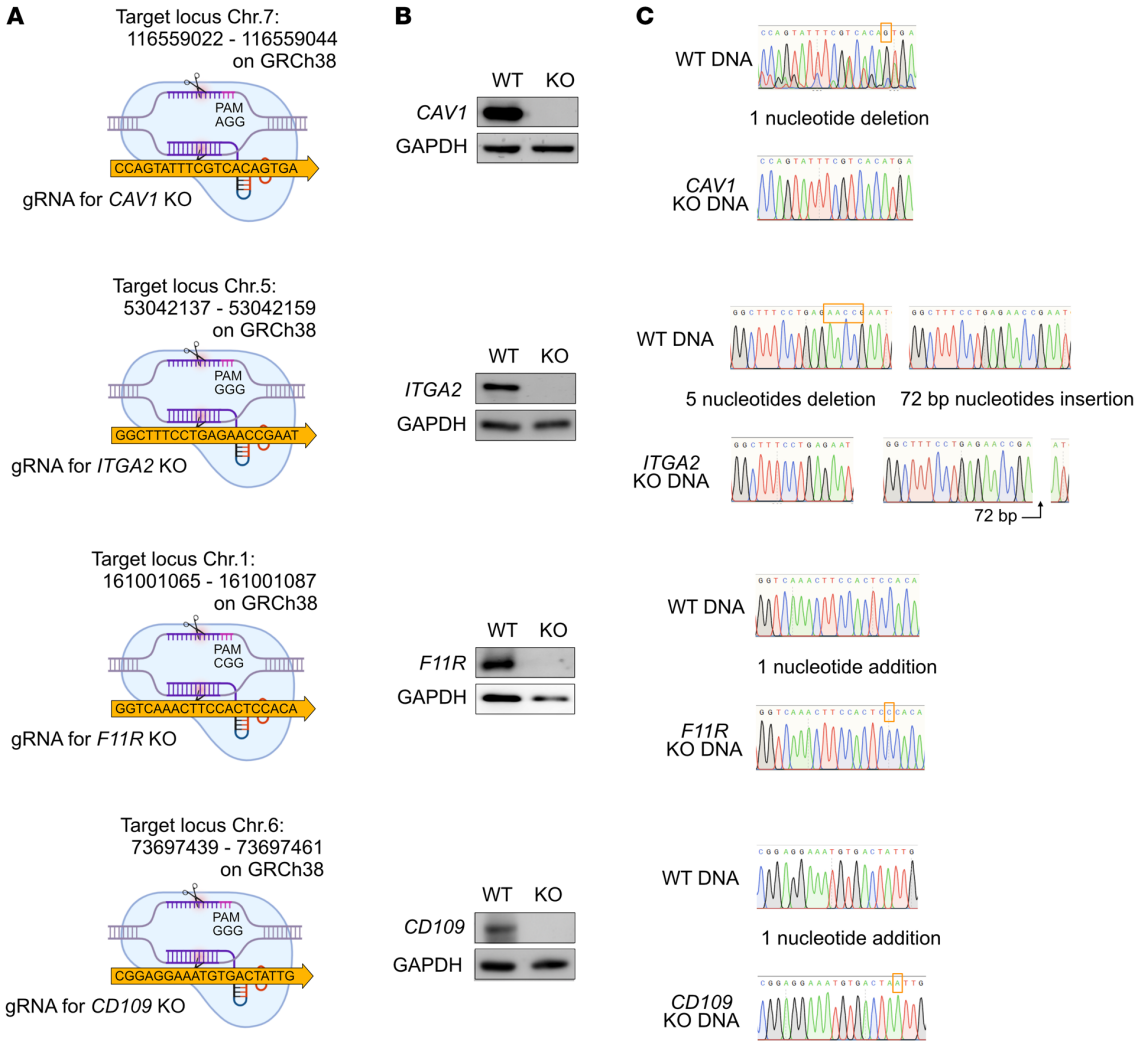
Establishment and characterization of KO clones for the candidate proteins in HuARLT cells. (**A**) Schematic diagram of the gRNA sequence for caveolin-1 (*CAV1*), integrin α2 (*ITGA2*), *F11R*, and *CD109*. The gRNA-recognizing site is indicated as the CRISPR target sequence. (**B**) Western blot analysis of each KO protein. WT, WT HuARLT cell; KO, KO HuARLT cell. (**C**) Target sequence analysis in WT and KO HuARLT cells. The PCR products containing the gRNA targeting region from the genomic DNA of WT and KO cells were cloned into a T-vector. The sequences of 10 colonies were analyzed by Sanger sequencing. The mutated region is indicated by a box.

To investigate whether the candidate proteins are associated with enhanced infection of senescent cells by KSHV, we performed KO of these proteins in HuARLT cells using CRISPR/Cas9 technology. Two single-guided RNAs (sgRNAs) for each target protein were used, and the most effective one was then selected (Figure 5A). Each clone was then isolated by limiting dilution, and the KO clone was chosen based on the target protein expression by Western blotting (Figure 5B). To analyze the mutated sequences in these clones, we cloned the PCR products, which contained the sgRNA-targeted region, into a T-cloning vector. Subsequently, we isolated 10 separate colonies and analyzed their sequences by Sanger sequencing. For *CAV1*, we found that all colonies exhibited a single nucleotide deletion at the Cas9-targeting site (Figure 5C). In the case of *ITGA2*, 8 colonies had a 5-nucleotide deletion and 2 colonies had a 72-bp insertion at the Cas9-targeting site (Figure 5C), indicating that 2 alleles had different mutations. For both *F11R* and *CD109*, all colonies demonstrated the addition of a single nucleotide at the Cas9-targeting site (Figure 5C). Although cell proliferation rates of *ITGA2* and *CD109* KO cells did not change, those of *CAV1* and *F11R* KO cells were significantly reduced compared with the WT cells (Supplemental Figure 10A). To examine whether senescence could still be induced in the KO cells, we withdrew doxycycline from HuARLT cells and then performed the SA-β-gal assay and assessed p21 expression. We observed SA-β-gal staining and p21 expression following the induction of senescence in all types of KO cells, which was comparable to that seen in WT cells (Supplemental Figure 10, B and C).

### Identification of crucial host factors for KSHV infection using KO cell clones.

**Figure 6:**
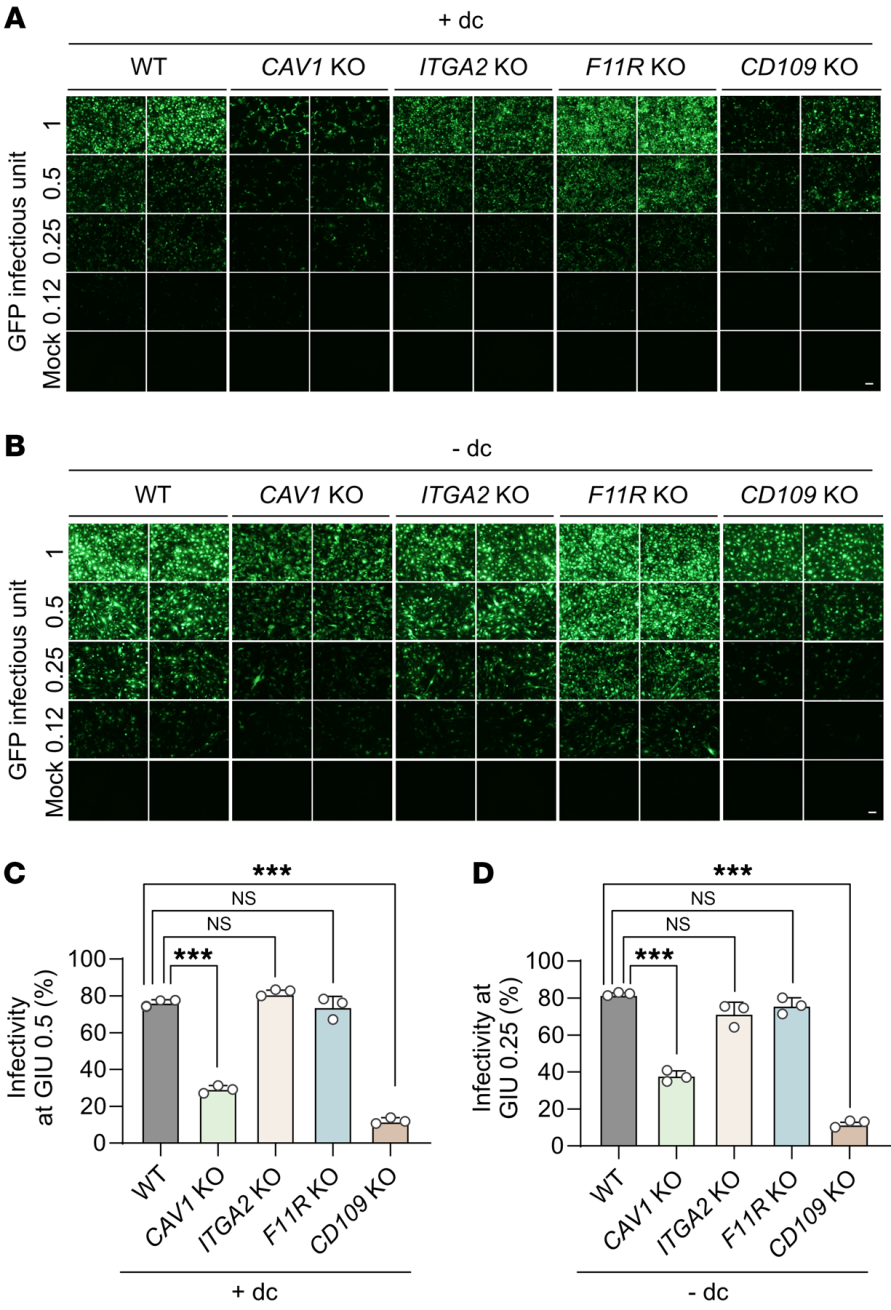
Analysis of KSHV infectivity in KO clones of *CAV1*, *ITGA2*, *F11R*, and *CD109*. An equivalent quantity of KSHV was used to infect the same number of WT and KO HuARLT cells, with or without induction of senescence. KSHV was prepared as GFP infectious units of 1 to infect approximately 90% of nonsenescent WT cells, followed by the infection of a 2-fold serially diluted virus into each group of conditioned cells. KSHV infectivity was measured using GFP expression. (**A** and **B**) Fluorescence microscopic visualization of KSHV-infected cells for each KO clone in nonsenescent (+dc, **A**) and senescent (–dc, **B**) HuARLT cells. *CAV1*, caveolin-1; *ITGA2*, integrin-α_2_. Scale bar: 100 μm. (**C** and **D**) Flow cytometric analysis of KSHV-infected cells for each KO clone in nonsenescent (+dc, **C**) and senescent (–dc, **D**) HuARLT cells. At the indicated GFP infectious units (GIU), the percentage of KSHV-infected cells was compared among each group. Data are representative of 3 independent experiments. Data are shown as mean ± SD. *n* = 3. ^***^P < 0.001, Dunnett’s test for multiple comparisons.

To evaluate the role of each candidate protein in KSHV infection, we infected the WT and KO cells, with or without induction of senescence. We employed 1 GFP infectious unit, which infected approximately 90% of nonsenescent WT cells. Additional cells were infected with 2-fold serially diluted virus. Similar to the results shown in Figure 1, senescent WT cells demonstrated higher viral infectivity than the nonsenescent cells (Figure 6, A and B). KO of *CAV1* and *CD109* inhibited KSHV infection in both nonsenescent and senescent cells. These results were confirmed by flow cytometric analysis (Figure 6, C and D, and Supplemental Figure 11). In contrast, KO cells for *ITGA2* and *F11R* did not affect viral infectivity (Figure 6, A–D). We observed a sigmoid pattern of infectivity with serially diluted viruses (Supplemental Figure 11). At 1 GFP infectious unit, nonsenescent WT cells had an approximately 90% infection rate, which was reduced to less than 50% following *CAV1* KO. Senescent cells also exhibited a significant difference in infectivity between WT and *CAV1* KO cells (95% vs 76%). At lower GFP infectious units (0.5 and 0.25), both nonsenescent and senescent cells exhibited a significant difference in KSHV infectivity between WT and KO cells (Figure 6, C and D). *CD109* KO cells presented a notable difference in infectivity compared with WT cells with or without induction of senescence (Figure 6, C and D). In contrast, KO of *ITGA2* and *F11R* did not affect infectivity in both senescent and nonsenescent cells at all the GFP infectious units tested (Figure 6, C and D, and Supplemental Figure 11). Taken together, these results indicate that caveolin-1 and CD109 are potential factors regulating KSHV infectivity. Importantly, we detected higher expression levels of both caveolin-1 and CD109 in replicative senescent HUVECs than nonsenescent cells (Supplemental Figure 12). Results of several previous studies demonstrated that caveolin-1 expression significantly increased with age in vivo and in vitro, which are consistent with our observations (17–19). While the relationship between aging and caveolin-1 expression is well established, emerging evidence also supports an age-dependent regulation of CD109 expression in vivo. Transcriptomic analyses across multiple tissues in *Macaca fascicularis* revealed consistent age-associated increases in *CD109* RNA expression, which were particularly prominent in endothelial cells, with the exception of retinal tissue. Although human studies were more limited in scope, transcriptional profiling of skin fibroblasts and HUVECs demonstrated similar age-dependent elevation of *CD109* expression (Table 2). Mechanistic insights into this age-associated regulation have emerged from studies of cellular senescence. Notably, human mesenchymal stem cells lacking the anti-aging gene *WRN* exhibit increased *CD109* expression concurrent with cellular senescence. The causality of this relationship was further supported by the observation that treatment with quercetin, an anti-aging compound, reduced *CD109* expression by 23% in these senescent cells (20). To validate these findings in human tissues, we performed immunohistochemical analyses comparing endothelial CD109 expression between neonatal and elderly tissue specimens. Our results demonstrated significantly elevated CD109 expression in aged endothelial cells (Supplemental Figure 13), providing direct evidence for age-associated CD109 upregulation in vivo. These findings, supported by both molecular and histological evidence, established CD109 as an age-regulated protein with potential implications for age-associated KSHV susceptibility.

**Figure 7:**
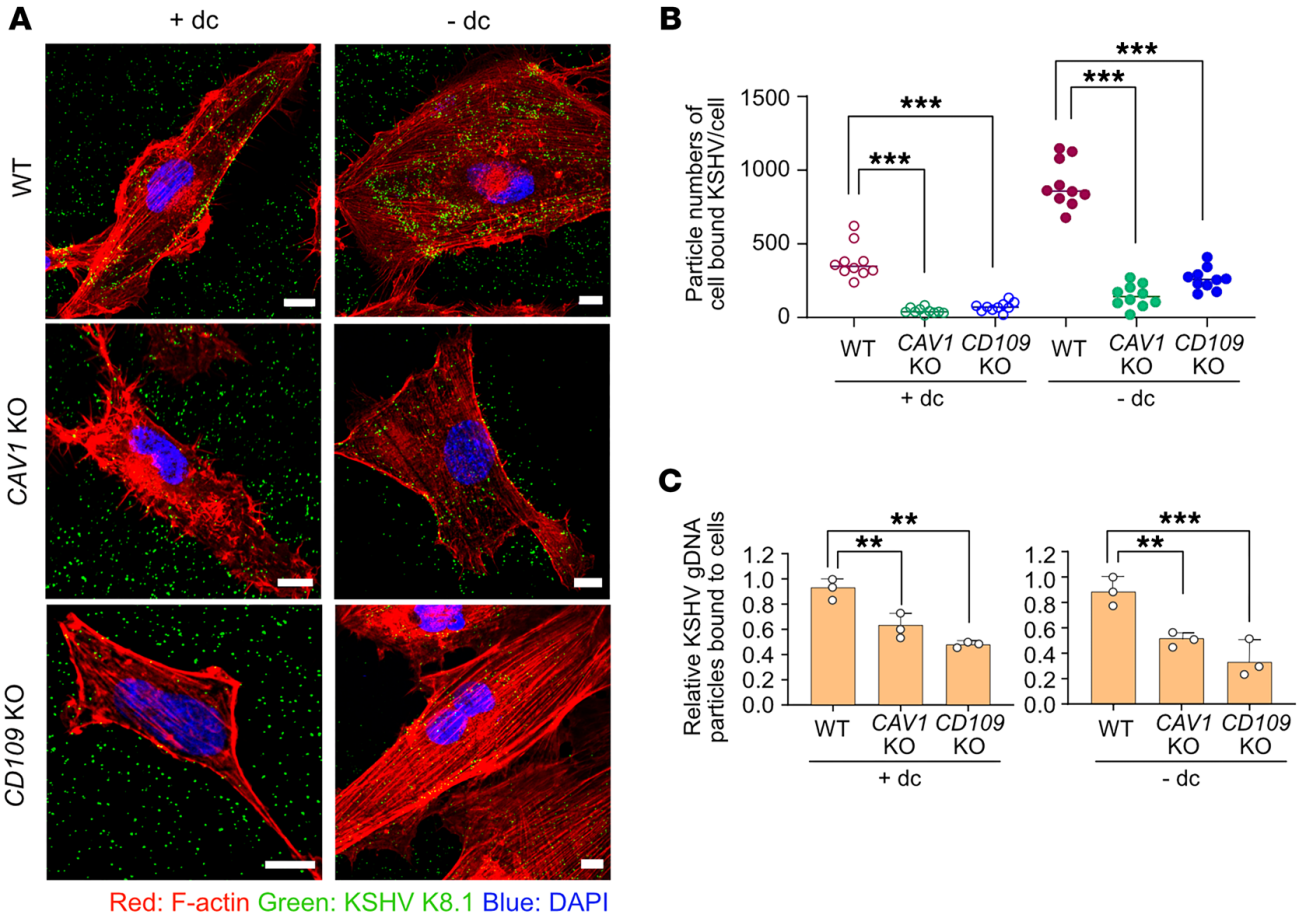
KSHV binding to the cell surface of *CAV1*-KO and *CD109*-KO HuARLT cells. (**A**) Representative confocal images of KSHV virus particles in *CAV1*-KO and *CD109*-KO HuARLT cells. Scale bars: 10 μm. (**B**) Analysis of the number of KSHV particles that bind to the cell surface per cell in the images in **A** and Supplemental Figure 13. Data are representative of 10 independent experiments. Data are shown as mean ± SD. *n* = 10. ^***^P < 0.001, Dunnett’s test for multiple comparisons. (**C**) Quantification of the cell-surface–bound KSHV genome in the KSHV-infected cells by quantitative PCR. After 1 hour of KSHV infection, the cells were immediately scraped, and genomic DNA extracted after washing. The KSHV genome was quantified in the extracted DNA by quantitative PCR using primers for KSHV ORF26. Data are representative of 3 independent experiments. Data are shown as mean ± SD. *n* = 3. ^**^P < 0.01; ^***^P < 0.001, Dunnett’s test for multiple comparisons.

To further confirm the roles of caveolin-1 and CD109 in KSHV infectivity, we examined binding of KSHV virions to the cell surface by staining for KSHV K8.1 proteins in *CAV1* and *CD109* KO cells (Figure 7 and Supplemental Figure 14). We observed viral particles on the cell surfaces of both nonsenescent and senescent WT cells, with significantly more particles observed on senescent cells (Figure 7, A and B). However, KO of either *CAV1* or *CD109* significantly decreased the number of viral particles on the cell surfaces. Similarly, KO of either *CAV1* or *CD109* also reduced genomic DNA of KSHV virions on the cell surface compared with WT cells (Figure 7C).

### Assessment of KSHV interaction with CD109 and caveolin-1.

**Figure 8:**
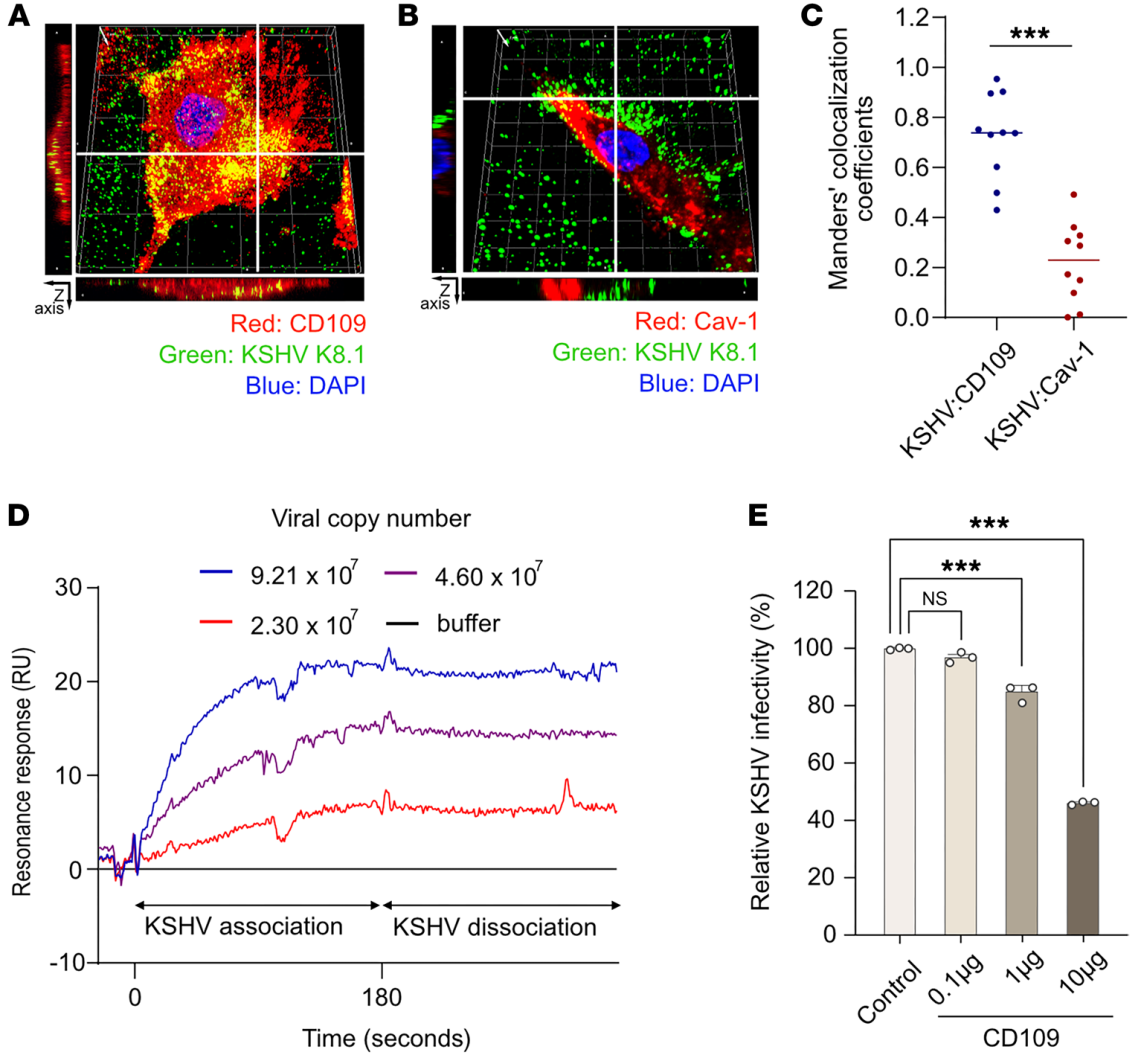
Analysis of KSHV interaction with caveolin-1 and CD109. (**A** and **B**) Three-dimensional confocal microscopy images of the colocalization of KSHV with CD109 (**A**) or caveolin-1 (Cav-1) (**B**). Senescent HuARLT cells infected with KSHV for 1 hour were stained with KSHV K8.1 antibody and target proteins, and *Z*-axis scanning was performed at 4 μm intervals, generating more than 10 scans. The bar-shaped images at the edges represent cross-sections, guided by white solid lines in the stacked image. (**C**) Quantitative analysis of the colocalization of KSHV with CD109 or caveolin-1 by Manders’ colocalization coefficient in KSHV-infected senescent HuARLT cells. *n* = 10. ^***^P < 0.001 (**D**) SPR analysis of KSHV binding to immobilized recombinant CD109. Data are representative of 3 independent experiments. (**E**) Neutralization of KSHV infectivity using a recombinant CD109 protein. Data are representative of 3 independent experiments. Data are presented as mean ± SD. *n* = 3. ^***^P < 0.001, Dunnett’s test for multiple comparisons.

We further examined whether KSHV virions directly bind to CD109 and caveolin-1 during infection of senescent HuARLT cells (Figure 8, A–C). Confocal microscopy confirmed that KSHV virions revealed by KSHV K8.1 protein staining were distributed on the cell surface and colocalized with CD109 but not caveolin-1 on the HuARLT cell surface within 1 hpi (Figure 8, A and B, and Supplemental Figure 15). The calculated Manders’ colocalization coefficients also support these findings (Figure 8C). These results indicate that K8.1 protein on KSHV virions directly binds to CD109 but not caveolin-1 on the cell surface. To confirm the role of CD109 in mediating binding of KSHV virions to the cell surface during infection, we established stable CD109 and caveolin-1 cell lines in 293T cells (Supplemental Figure 16, A and B). KSHV infectivity was increased in cells overexpressing CD109 but not caveolin-1 (Supplemental Figure 16, C and D). As expected, overexpression of CD109 but not caveolin-1 enhanced the cell surface binding of KSHV genomic DNA (Supplemental Figure 16E). To confirm the direct binding of KSHV virions to CD109 protein, a recombinant CD109 protein was immobilized on the sensor chip for surface plasmon resonance (SPR) analysis. KSHV virions bound to CD109-immobilized sensor chip in a dose-dependent manner when introduced at 3 different concentrations in the running buffer (Figure 8D). Furthermore, when recombinant CD109 and KSHV virions were mixed and used for infection, we observed a decrease in KSHV infection rate proportional to the amount of recombinant CD109 added (Figure 8E). These findings indicate that recombinant CD109 inhibits KSHV infection by blocking bindings of KSHV virions to the cell surface. Hence, KSHV virions directly bind to CD109 during entry into cells, and the enhanced expression of CD109 in senescent cells promotes KSHV entry and infection.

### CD109 specifically interacts with KSHV gH/gL glycoproteins.

**Figure 9:**
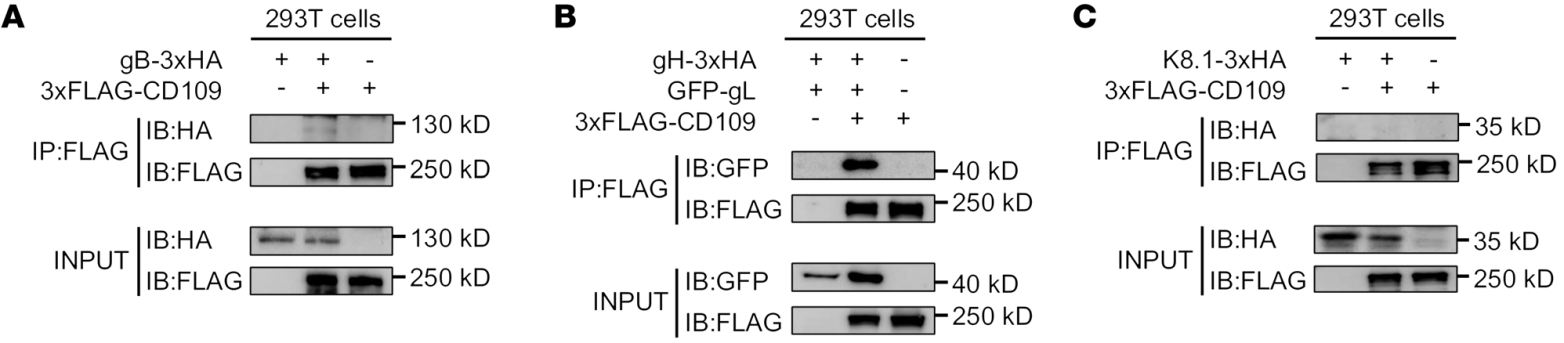
KSHV gH/gL interacts with CD109. Co-IP analysis of CD109 with KSHV glycoproteins gB (**A**), gH/gL (**B**), and K8.1 (**C**). HEK-293T cells were cotransfected with the indicated plasmids for 24 hours, after which cell lysates were subjected to IP using anti-FLAG magnetic agarose beads. The resulting complexes were then analyzed by immunoblotting (IB) using the indicated antibodies. Data are representative of 3 independent experiments.

To identify the specific viral glycoproteins mediating the interaction with CD109, we generated KSHV glycoproteins tagged with either 3xHA (gB, gH, K8.1) or GFP (gL). Coimmunoprecipitation (co-IP) analyses revealed that the gH/gL heterodimer specifically interacts with CD109, while no binding was detected with either gB or K8.1 glycoproteins (Figure 9). This finding is consistent with the SPR results showing direct binding between CD109 and intact KSHV virions, and specifically identifies the gH/gL complex as the viral binding partner for CD109.

## Discussion

In this study, we have found that senescent human endothelial cells are more susceptible to KSHV infection. This heightened susceptibility is associated with the increased expression of CD109 and caveolin-1 proteins in senescent cells compared with their nonsenescent counterparts. In particular, CD109 directly mediates the binding of KSHV virions to the cell surface during infection. The virus enters host cells through a multistep process involving interactions between viral envelope glycoproteins and host cell-surface receptors. Several host cell-surface receptors have been identified as potential entry receptors for KSHV (16). Integrins are transmembrane receptors responsible for facilitating cell adhesion and signaling processes. They have been shown to interact with KSHV envelope glycoprotein gB (21, 22). HSPGs are glycosaminoglycan chains that are attached to cell-surface proteoglycans and have been shown to interact with glycoproteins K8.1 and gH alone, and the gH/gL complex (23). EphA receptors are a family of transmembrane proteins involved in cell signaling and have been shown to interact with the gH/gL complex (24, 25). Our identification of the direct interaction between CD109 and the gH/gL glycoprotein complex provides insights into mechanisms of KSHV entry into cells. Our results show that the gH/gL heterodimer uses CD109 as a binding receptor in addition to the previously identified HSPGs and EphA, suggesting the complexity of KSHV cell-entry mechanisms. This raises intriguing questions about how these distinct receptor-glycoprotein interactions might cooperate to facilitate KSHV infection. Further investigation of the potential interplay among CD109, HSPGs, EphA receptors, and the gH/gL complex will be crucial for a full understanding of the molecular mechanisms of KSHV entry into cells.

Our study demonstrated critical roles of CD109 and caveolin-1 in KSHV infection. Caveolin-1 is a membrane protein, and most of its protein structures are embedded within the cell membrane (26), making it impossible for it to directly interact with KSHV virions on the cell surface. Our findings indicated that overexpressing caveolin-1 did not lead to increased KSHV infectivity, implying that KSHV virions are unlikely to directly bind to caveolin-1 on the cell surface. However, KSHV infectivity significantly decreased in caveolin-1 KO cells, suggesting that caveolin-1 is an essential factor for KSHV infection by regulating other step(s) of KSHV infection. Caveolin-1, while renowned for its diverse cellular functions including signal transduction, lipid homeostasis, and tumor suppression, plays an indispensable role in caveolin-mediated endocytosis. Previous studies have established that KSHV can enter cells through multiple pathways, depending on the cell type. In human dermal microvascular endothelial cells (HMVEC-d), studies have demonstrated that KSHV primarily uses macropinocytosis as its major route of cell entry (27), whereas lipid rafts play a critical role in KSHV infection in these cells (28). Greene and Gao demonstrated that in HUVECs, KSHV cell entry is involved with clathrin-mediated endocytosis (15). Additionally, in monocytic THP-1 cells, caveolin-mediated endocytosis has also been implicated in KSHV cell entry (29). Our study adds to this complex picture of KSHV internalization in endothelial cells. The interaction between CD109 and caveolin-1, as reported by Bizet et al. (30), suggests a potential mechanism by which the caveolar pathway might facilitate KSHV cell entry. Given the role of caveolin-1 in KSHV infection in this study, it is possible that CD109 acts as a receptor, potentially concentrating virions near caveolae or facilitating their internalization via the caveolar pathway. This hypothesis is consistent with our observation of increased KSHV infectivity in senescent cells, which have increased levels of both CD109 and caveolin-1. Our findings suggest a potential link of CD109 to the caveolar endocytic pathway during KSHV cell entry, but the exact role of CD109 in directing KSHV virions to specific internalization routes needs to be clarified.

CD109 is a cell-surface glycoprotein expressed in various cell types (31), including endothelial cells (32). Its complex functions vary with cell type and cellular context. One of its roles is to regulate the TGF-β signaling pathway by binding to and blocking the TGF-β receptor, preventing downstream signaling (30, 33). Previous studies have demonstrated that KSHV modulates TGF-β signaling through multiple mechanisms: KSHV-encoded LANA inhibits TGF-β signaling by binding to the promoter region of the TGF-β type II receptor (34), and KSHV miRNAs directly target TGF-β pathway components (35–37). Given the pro- and antitumorigenic effects of TGF-β in tumor progression (38, 39), further studies are needed to elucidate whether the KSHV-CD109 interaction represents an additional mechanism by which KSHV regulates TGF-β signaling during infection and KS development, particularly in the context of cellular senescence and aging.

Aging is generally associated with an increase in the number of senescent cells in various tissues (40). This can lead to a gradual decline in immune function, making older individuals more susceptible to certain viral infections (41). However, the association between senescence and viral infection may be more complex than expected. The interplay between senescence and viral infection is influenced by several factors, including the virus type, host cell characteristics, and the specific infection context. While senescence can act as a host defense mechanism against viral infections, some viruses have evolved mechanisms to trigger senescence in host cells as a strategy to enhance viral infection (42, 43). Previous research has demonstrated that KSHV can alter cellular senescence in KSHV-infected cells through certain viral proteins (44–47). Based on previous studies (44, 47), senescence might serve an antiviral or anticancer function during KSHV infection. Contrary to this notion, we have shown that KSHV exploits senescent endothelial cells to enhance lytic replication and virus production, rather than bypassing senescence (Supplemental Figure 4). This finding is particularly relevant in the context of KS, where a small number of cells undergoing spontaneous lytic replication are considered critical for KSHV pathogenesis (48). Thus, senescent cells may serve as a source of lytic replication among KSHV-infected cells, thereby contributing to the progression of KSHV-associated cancers. While the precise mechanism of enhanced KSHV lytic replication remains to be defined, it could be speculated that this might be due to the high level of oxidative stress in the senescent cells (49). In fact, previous studies have shown that oxidative stress can promote KSHV lytic replication (50–52).

Beyond cellular senescence, our investigation of CD109 expression extends to HIV infection, where multiple independent studies suggest an association between HIV status and CD109 upregulation. Transcriptional analysis of HIV-1 latency models, encompassing both established cell lines (CA5, EF7, CG3) and primary T cells isolated from HIV-infected patients (JWEAU-A10 and JWEAU-C6), revealed significantly elevated CD109 gene expression compared with their uninfected counterparts (53). This relationship is further supported by quantitative glycoproteomic profiling demonstrating a striking 30.5-fold increase in CD109 protein expression in HIV-1–infected ACH-2 cells compared with their uninfected parental A3.01 cells (54). A recent large-scale plasma proteomics analysis of 1,293 proteins identified CD109 among the top 10 proteins that correlated with cell-associated HIV-1 DNA levels, highlighting its potential role in HIV pathogenesis (55). These convergent findings from diverse experimental approaches suggest a robust biological link between HIV infection and CD109 expression, potentially contributing to the increased KSHV susceptibility observed in HIV-positive individuals.

The present study was the first, to our knowledge, to illustrate the impact of senescence on KSHV infection in endothelial cells. Nevertheless, this intriguing observation raises several questions regarding KSHV-infected senescent endothelial cells. Senescent cells typically bolster their antiviral response by upregulating specific genes associated with innate immunity. Thus, the unexpected increase in KSHV infectivity in senescent endothelial cells warrants further investigation. It would be valuable to explore alterations in the immune response to KSHV in these senescent cells. In addition, senescence has been implicated in cancer development and metastasis/dissemination by altering the tumor microenvironment (56, 57). Hence, examining cellular differentiation and tumor development in senescent lymphatic endothelial cells or mesenchymal stem cells could present an intriguing avenue for investigating KSHV-induced tumorigenesis.

In conclusion, our study identified host factors that facilitate KSHV entry and trafficking in human endothelial cells, particularly in senescent cells. These findings provide insights into the mechanisms underlying KSHV pathogenesis related to aging and reveal an alternative infection pathway for KSHV entry into human endothelial cells. Understanding these mechanisms may contribute to the development of targeted therapeutic strategies for KSHV-associated malignancies, particularly in the aging and immunocompromised populations.

## Methods

### Sex as a biological variable.

We analyzed CD109 expression in human tissue samples from both young (placenta and umbilical cord) and elderly individuals. Although samples were collected from both males and females, sex was not considered as a biological variable.

### Cell culture and reagents.

HuARLT cells, generated in house (11), were cultured in endothelial cell growth medium-2 (Promocell) with supplements and 2 μg/mL doxycycline (Sigma-Aldrich). HUVECs purchased from Promocell were cultured in endothelial cell growth medium-2 with supplements. All HUVECs used in this experiment underwent fewer than 10 passages, except for those subjected to the induction of replicative senescence. Lenti-X 293T cells were purchased from TaKaRa and cultured in high glucose DMEM supplemented with 10% FBS (GeneDEPOT) and 1% antibiotics (Gibco, Thermo Fisher Scientific). All cells were cultured in a humidified atmosphere containing 5% CO_2_ at 37°C. The senescence of HuARLT cells was induced by culturing without doxycycline for 5 days, whereas the senescence of HUVECs was induced by treating the cells with 50 nM doxorubicin for 5 days. In both cases, subculturing was performed every 2 days while senescence was induced. Conversely, replicative senescence was prompted by prolonged cultivation of early passage HUVECs. HUVECs were subcultured every 2 days until reaching passage 35.

### SA-β-gal staining assay.

Senescent HuARLT cells and HUVECs were seeded in 6-well plates at 1 × 10^5^ cells per well. The Senescence β-Galactosidase Staining Kit (Cell Signaling Technology) was used according to the manufacturer’s instructions. The stained cells were visualized and images captured using a TS100 inverted-phase microscope (Nikon). To quantify cells positive for SA-β-gal using flow cytometry, we utilized the CellEvent Senescence Green Flow Cytometry Assay Kit (Thermo Fisher) to stain senescent HuARLT cells and HUVECs following the manufacturer’s instructions. Stained cells were analyzed using a Guava Flow Cytometer (Luminex).

### Analysis of cell proliferation.

To evaluate cell proliferation in both nonsenescent and aged endothelial cells, we employed the trypan blue exclusion assay to count HuARLT cells and HUVECs while inducing senescence. To determine the population doubling level (PDL) in replicatively senescent HUVECs, cell counts were performed daily during subculturing. The PDL was calculated using the following formula: PDL = initial PDL + 3.322 × (log[final cell count] + log[initial cell count]).

### Cell cycle analysis.

Cells were detached from culture using Trypsin-EDTA (Gibco) and counted using the trypan blue exclusion method. The cells were then adjusted to 5 × 10^5^ cells in culture medium and washed with 1 ml of PBS. The cells were fixed with ice-cold 70% ethanol and incubated overnight at –20°C. Following fixation, the cells were permeabilized with 0.25% Triton-X 100 for 30 minutes at 4°C. The cells were then stained with 20 μg/mL of propidium iodide (Sigma-Aldrich) and 10 μg/mL of RNase in PBS for 30 minutes at room temperature. The stained cells were analyzed using a Guava Flow Cytometer (Luminex).

### KSHV isolation and infection.

KSHV was isolated from iSLK BAC16 cells containing the recombinant KSHV BAC16 genome using a previously described method (58). KSHV infection of endothelial cells was achieved as described previously with some modifications (59). Briefly, senescent or nonsenescent cells were seeded in a 6-well plate at 2 × 10^5^ cells per well 1 day prior to infection. The cells were then washed with PBS and the medium replaced with Opti-MEM (Gibco, Thermo Fisher Scientific) containing the same amount of KSHV per group and 5 μg/mL polybrene (Santa Cruz Biotechnology). The plate was then centrifuged for 1 hour at 2,000*g* and 25°C. Finally, the virus-containing media was replaced with fresh culture medium.

### Analysis of KSHV-infected cells by fluorescence microscopy and flow cytometry.

The KSHV-infected cells were evaluated for the expression of GFP, which indicates infection. For imaging, cells expressing GFP were observed and photographed using an inverted fluorescence microscope (TS100, Nikon). Each test group was photographed 3 times, and representative data were presented. For flow cytometry, infected cells were detached using trypsin-EDTA (Gibco, Thermo Fisher Scientific), neutralized with endothelial cell growth medium-2 (Promocell), washed with 1 mL PBS, and resuspended in 500 μL PBS before analysis by a Guava Flow Cytometer (Luminex). Nonsenescent endothelial cells were used to titer the concentration of infectious KSHV. Experimentally, after establishing infectious titers that show about 10%–30% of GFP-positive cells in nonsenescent cells, the degree of KSHV infection was compared and analyzed in both nonsenescent and senescent cells by infecting them with the same titer of the virus. In the case of KSHV infection, comparative analysis in KO cells, with a titer showing about 90% infection in nonsenescent WT cells set as 1 GFP infectious unit, 2-fold serially diluted virus was prepared and used to infect each prepared cell.

### Quantification of viral genome or mRNA in KSHV-infected cells.

One day prior to KSHV infection, control and senescent cells were seeded in 6-well plates at 2 × 10^5^ cells per well. To quantify the internalized viral genomic DNA, 4 hpi, the cells were washed with PBS and then detached using trypsin-EDTA. The total DNA was extracted from the cells using the DNeasy kit (QIAGEN) and normalized to a consistent concentration per volume using NanoDrop One (Thermo Fisher). For analysis of viral mRNA expression, RNA was extracted from infected cells 1 day after infection using Ribospin (Geneall). The RNA concentration was standardized across samples by quantifying with NanoDrop. Subsequently, the standardized RNA was subjected to reverse transcription using TaKaRa 5X RT Master Mix (Takara). Real-time PCR was carried out with TaKaRa TBGreen Fast qPCR Mix (Takara). Amplification for human GAPDH was used as a housekeeping gene for normalization. The cycle conditions were 95°C for 30 seconds, followed by 40 cycles of 95°C for 5 seconds and 60°C for 10 seconds. The primers used in this experiment were KSHV *ORF26* gene, 5′-GGAGATTGCCACCGTTTA-3′ (sense) and 5′-ACTGCATAATTTGGATGTAGTC-3′ (anti-sense); KSHV ORF71 gene, 5′-GGATGCCCTAATGTCAATGC-3′ (sense) and 5′-GGCGATAGTGTTGGGAGTGT-3′ (anti-sense); and *GAPDH*, 5′-GGTATGGTGGAAGGACTC-3′ (sense) and 5′-GTAGAGGCAGGGATGATG-3′ (anti-sense), which were synthesized at Genotech. Specificity of the primers was confirmed by melting curve analysis.

### Immunofluorescence and confocal image analysis.

To analyze the binding of KSHV to the cellular surface, we employed a modified method based on Bottero et al. (60). In the confocal microscopy assay, 1 × 10^5^ cells were seeded on coverslips within a 24-well plate. The following day, each sample was infected with an equivalent volume of KSHV as described above. After the infection process, the infected cells were washed 3 times with PBS and immediately fixed with 4% paraformaldehyde for 30 minutes. With the exception of permeabilization steps, the sample preparation for confocal microscopy followed the immunofluorescence assay (IFA) procedure (58, 61). Stained coverslips were mounted onto glass slides and analyzed using a ZEISS LSM 880 confocal microscope (ZEISS), with subsequent analysis by ZEISS Zen Blue Edition software. Particles were quantified using Nikon NIS software (Nikon). For quantifying KSHV particles internalized by cells after infection, cells were incubated under 5% CO_2_ at 37°C for 4 hours. Further staining was performed as described in the IFA method (58, 61). Stained cells were visualized by an E300 fluorescence microscope (Nikon). Images and particle counts were analyzed by Nikon NIS software (Nikon). Mouse anti-KSHV K8.1 antibody (1:100 dilution; SC-65446; 4A4; Santa Cruz Biotechnology), rabbit anti-caveolin-1 (1:100 dilution; A1555; Abclonal), rabbit anti-CD109 (1:100 dilution; 486955; E8L2W; Cell Signaling Technology), rat anti-LANA (1:100 dilution; Ab4103; LN53; Abcam), and mouse anti-KSHV ORF 65 (1:100 dilution) (62) were used as primary antibodies. Alexa Fluor 568 phalloidin (Thermo Fisher) was used to stain F-actin. Alexa Fluor 568–conjugated anti-rat IgG (1:100 dilution; A11077; Thermo Fisher), Alexa Fluor 488–conjugated anti-mouse IgG antibody (1:100 dilution; A11001; Thermo Fisher), and Alexa Fluor 568–conjugated anti-mouse IgG antibody (1:100 dilution; A11004; Thermo Fisher) were used as secondary antibodies. Tris-buffered saline with 0.1% Tween 20 was used for the washing process after the antibody treatment. Nuclei were stained with DAPI. In the case of DAPI and phalloidin, they were used after secondary antibody treatment. To analyze colocalization, we performed quantitative colocalization analysis using Fiji software and applied the Colocalization Threshold plugin to calculate the Manders’ colocalization coefficient (R). A maximum coefficient value of “1” indicates perfect overlap, whereas a minimum value of “0” represents complete exclusion.

### Analysis of copy number of KSHV genome.

The counting of KSHV genome copy number was conducted using a previously described method (63). One day prior to infection, 2 × 10^5^ cells were seeded into each well of a 6-well plate. Following the procedure outlined above, the cells were infected. One day after infection, the cells were washed twice with PBS and then cultured under the desired conditions. The supernatant containing KSHV particles was collected after the culture period for a set duration. This supernatant was treated with RQ1 RNase-Free DNase (Promega) according to the manufacturer’s instructions to remove free DNA, and the DNA within the virion was extracted using the DNeasy Kit (QIAGEN). For quantification, a standard was established using pUC19 containing KSHV *ORF26*. The target sample and 10-fold serial diluted standards were quantified using the TaKaRa TBGreen Fast qPCR Mix (Takara), following the aforementioned procedure. The DNA quantity was determined using a series of standards with an *R^2^* value over 0.99.

### Quantitative PCR analysis of KSHV binding and entry into host cells.

The modified method previously described by Bottero et al. (60) was used to determine the relative binding and entry of KSHV virions. HuARLT cells and HUVECs were seeded in a 6-well plate at 2 × 10^5^ cells per well. To assess KSHV binding to host cells, they were thoroughly washed with PBS 3 times after infection and detached by scraping. The detached cells were collected by centrifugation at 800*g* and washed twice with PBS to eliminate any unbound viral particles. To evaluate KSHV entry into host cells, cells were seeded in 6-well plates at a density of 2 × 10^5^ cells per well. The next day, the cells were infected with KSHV and then incubated under 5% CO_2_ at 37°C for 4 hours. Following incubation, cells were washed 3 times with PBS and treated with trypsin-EDTA to detach the cells and eliminate any remaining virus. The detached cells were then washed with PBS in 2 additional centrifugation steps. DNA extraction and quantification of the KSHV genome were performed as described above.

### SDS-PAGE, in-gel digestion, and sample preparation for LC-MS/MS.

To prepare protein samples for analysis, 30 μg of each sample were separated by 12% SDS-PAGE and stained with Coomassie Brilliant Blue R-250. The entire gel was then divided into 8 sections according to the molecular weights of the proteins. The sliced gels were digested with trypsin (Promega) for 16 hours at 37°C after reduction with 10 mM dithiothreitol and alkylation of cysteines with 55 mM iodoacetamide. The digested peptides were recovered by an extraction solution containing 50 mmol/L ammonium bicarbonate, 50% acetonitrile, and 5% trifluoroacetic acid. The extracted tryptic peptides were dissolved in 0.5% trifluoroacetic acid prior to further fractionation by LC-MS/MS.

### LC-MS/MS analysis.

Tryptic peptide samples were loaded onto a 2G-V/V trap column (Waters) for the enrichment of peptides and removal of chemical contaminants. Concentrated tryptic peptides were eluted from the column and directed onto a 10 cm × 75 μm inner diameter (i.d.) C18 reverse phase column (PROXEON) at a flow rate of 300 nL/min. Peptides were eluted using a gradient of 0%–65% acetonitrile for 80 minutes. All MS and MS/MS spectra were acquired in a data-dependent mode using an LTQ-Velos ESI Ion Trap mass spectrometer (Thermo Fisher). Every complete MS scan (*m/z* range 300 to 2,000) focused on the most abundant precursor ions in the MS spectra. For protein identification, MS/MS spectra were analyzed by MASCOT (Matrix Science, http://www.matrixscience.com). The human protein sequence database (UniProt_proteome_hu) was downloaded from UniProt and used for protein identification. Mass tolerance of the parent or fragment ion was 0.8 Da. Carbamidomethylation of cysteine and oxidation of methionine were considered to be variable modifications of tryptic peptides in the MS/MS analysis.

### Flow cytometry.

Nonsenescent and senescent cells were detached by trypsin-EDTA. The detached cells were reacted with primary antibody for 30 minutes at 4°C. The primary antibodies used were rabbit anti-xCT (1:100 dilution; A13685; Abclonal), anti-ephrin A2 (1:100 dilution; A23177; Abclonal), anti–HO-1 (1:100 dilution; A1346; Abclonal), anti–caveolin-1 (1:100 dilution; A1555; Abclonal), anti–integrin α2 (1:100 dilution; A7629; Abclonal), anti-P4HB (1:100 dilution; A0692; Abclonal), anti-F11R (1:100 dilution; A1241; Abclonal), anti-VASP (1:100 dilution; A8862; Abclonal), anti-CD109 (1:100 dilution; 486955; E8L2W; Cell Signaling Technology), anti–integrin α_3_β_1_ (1:100 dilution; bs-1057R; Bioss), anti–integrin α_V_β_3_ (1:100 dilution; bs-1310R; Bioss), anti–integrin α_V_β_5_ (1:100 dilution; bs-1356R; Bioss); and mouse anti-CD98 (1:100 dilution; sc-59145; 4F2; Santa Cruz). Following the reaction, the cells were washed twice with PBS and a secondary antibody was added. The secondary antibodies used in this experiment were mouse F(ab)2 IgG (H+L) APC-conjugated antibody (10 μl/10^6^ cells; F0101B, R&D systems) and rabbit IgG APC-conjugated antibody (10 μl/10^6^ cells; F0111, R&D systems). The cells were analyzed using a Guava Flow Cytometer (Luminex).

### Establishment of KO clones.

KO clones were obtained using the CRISPR/Cas9 system following previously described methods with minor modifications (61, 64). Briefly, TrueGuide synthetic sgRNA (Thermo Fisher) was used for guide RNAs (gRNAs) against target genes: CRISPR639819_SGM (CCAGTATTTCGTCACAGTGA) for *CAV1*, CRISPR956294_SGM (GGCTTTCCTGAGAACCGAAT) for *ITGA2*, CRISPR830351_SGM (GGTCAAACTTCCACTCCACA) for *F11R*, andCRISPR652545_SGM (CGGAGGAAATGTGACTATTG) for *CD109*. Each sgRNA and Truecut Cas9 protein, version 2 (Thermo Fisher), was transfected into HuARLT cells using Lipofectamine CRISPRMAX/Cas9 transfection reagent (Thermo Fisher) following the manufacturer’s instructions. After 2 days, the transfected cells were harvested, the cell concentration adjusted to 8 cells/mL, and the cells seeded into a 96-well plate at 100 μL/well to isolate a single clone. The sequencing analysis of DNA from the KO clones followed the same method as described previously (61, 64). To analyze the sequences of each target gene, T-vector cloning was used with the following primers: CAV1 _S, 5′- TTCCCAAGTTCCAAGTGATGC-3′ (sense) and CAV1_AS, 5′-GGAATAGACACGGCTGATGC-3′ (anti-sense) for *CAV1*; ITGA2_S, 5′-AAAGGCAGCAGGTCAAATCA-3′ (sense) and ITGA2_AS, 5′-AGATGCGTGGAAAATGTGCTA-3′ (anti-sense) for *ITGA2*; F11R-S, 5′-AGTGCACTCTTCTGAACCTGA-3′ (sense) and F11R_AS, 5′-CTCATGGCCTTCCTACCCC-3′ (anti-sense) for *F11R*; and CD109_S, 5′-TAACACAGGCCTTCCTCTAGT-3′ (sense) and CD109_AS, 5′-ACCCTTCTGCAGTAATAACTGG-3′ (anti-sense) for *CD109*. The reference sequences for *CAV1*, *ITGA2*, *F11R*, and *CD109* were based on specific regions of *Homo sapiens* chromosome 5 (GRch38 primary assembly; 116,524,785 to 116,561,185; Entrez Gene ID 857), chromosome 5 (GRch38 primary assembly; 52,989,326 to 53,094,779; Entrez Gene ID 3673), chromosome 1 (GRch38 primary assembly; 160,995,211 to 161,021,343; Entrez Gene ID 50848), and chromosome 6 (GRch38 primary assembly; 73,679,192 to 73,828,317; Entrez Gene ID 135228), respectively.

### Cloning.

To clone KSHV glycoproteins gB-3xHA, gH-3xHA, K8.1-3xHA, and GFP-gL, we first inserted a flexible linker (GGGGS) and 3xHA into the pcDNA3.1(+) vector using inverse PCR (iPCR) to create the pcDNA3.1-3xHA construct. KSHV genomic DNA was extracted from iSLK BAC16 cells and used as a template. The sequence- and ligation-independent cloning (SLIC) method was employed to insert gB, gH, and K8.1 into pcDNA3.1-3xHA, and gL was inserted into GFP-C1. First, pcDNA3.1-3xHA and each glycoprotein were amplified to generate linear fragments with 40 bp homologous sequences at both ends. Meanwhile, the GFP-C1 vector was linearized by digestion with EcoRI and BamHI restriction enzymes. Subsequently, the linearized PCR products or vector were mixed and incubated at room temperature for 2.5 minutes with T4 DNA polymerase (Enzynomics). Following this, the DNA mixtures were kept on ice for 10 minutes and then transformed into competent *E*. *coli* cells. We also engineered FLAG-CD109 purchased from Sino Biological into 3xFLAG-CD109 using iPCR. Prior to use, gB-3xHA, gH-3xHA, K8.1-3xHA, 3xFLAG-CD109, and GFP-gL underwent DNA sequencing analysis (Bionics) for verification.

### Co-IP assay.

To confirm the physical interaction between KSHV glycoproteins and the CD109 receptor, co-IP was performed. Briefly, 50 μL of anti-FLAG magnetic agarose beads (Pierce, A36797) slurry was mixed with 500 μL of lysis buffer. The beads were collected using a magnetic stand, and the supernatant was removed. This washing step was repeated 3 times. Next, 500 μg of cell lysate containing FLAG-tagged proteins was added to the beads and mixed, followed by incubation at room temperature for 20 minutes with occasional vortexing. After incubation, the beads were collected again and washed twice with 500 μL of PBS buffer and finally washed with 500 μL of purified water. To elute the immunoprecipitated complexes, 100 μL of 0.1M glycine buffer (pH 2.8) was added to the beads, which were incubated at room temperature for 5 minutes with frequent vortexing. The supernatant was collected, and 15 μL of 1M Tris buffer (pH 8.5) was added to neutralize the pH. The immunoprecipitated complexes were subsequently analyzed by Western blotting.

### Western blot analysis.

Western blot analysis was performed as described previously (63). The primary antibodies were mouse-derived anti-p16 (1:1,000 dilution; sc-56330, JC8; Santa Cruz), anti-p21 (1:1,000 dilution; sc-6246, F-5; Santa Cruz), anti-HHV-8 K8.1A/B (1:1,000 dilution; sc-65446; 4A4; Santa Cruz), anti-KSHV ORF45 (1:1,000 dilution; MA5-14769; 2D4A5; Thermo Fisher), anti-HA (1:1,000 dilution; 26183; Thermo Fisher), and anti-GFP (1:1,000 dilution; sc-9996; Santa Cruz). Anti-HO-1 (1:1,000 dilution; A19062; Abclonal), anti-caveolin-1 (1:1,000 dilution; A19006; Abclonal), anti-integrin α_2_ (1:1,000 dilution; A19068; Abclonal), anti-P4HB (1:1,000 dilution; A0692; Abclonal), anti-F11R (1:1,000 dilution; A1241; Abclonal), anti-VASP (1:1,000 dilution; A0166; Abclonal), anti-GAPDH (1:1,000 dilution; CSB-MA000071M0m; 14C2F11; Cusabio), anti-FLAG (1:1,000 dilution; F7425, Merck Millipore), and anti-CD109 (1:1,000 dilution; sc-271085; C-9; Santa Cruz) antibodies were all of rabbit origin. The secondary reaction antibodies were HRP-conjugated goat anti-mouse IgG heavy and light chain antibody (1:5,000 dilution; A90-116P; Bethyl Laboratories) and HRP-conjugated goat anti-rabbit IgG heavy and light chain antibody (1:5,000 dilution; A120-101P; Bethyl Laboratories). After all reactions, blots were captured by an Amersham ImageQuant 800.

### Establishment of stable cell lines expressing caveolin-1 and CD109.

To create cells that overexpress caveolin-1 and CD109, plasmids containing the specific target genes and control vectors were acquired from Sino Biological. The plasmid vector was subsequently enriched within the *E*. *coli* DH5α system. Next, transfection of the plasmid vector into Lenti-X 293T cells was achieved using the Lipofectamine 3000 (Thermo Fisher) in accordance with the manufacturer’s instructions. Selection of the transfected cells was performed using 20 μg/mL Hygromycin B (Thermo Fisher) over a span of 2 weeks. Prior to utilization, the success of the overexpression was validated by Western blotting.

### SPR analysis.

For SPR analysis, sensor chips comprising mixed self-assembled monolayers of 90% alkane-monothiol PEG3KOH and 10% alkane-monothiol PEG6K-COOH were mounted onto the instrument using immersion oil. The chip activation involved modifying the surface-free carboxyl groups by injecting a solution of 0.1 M 1-ethyl-3-(3-dimethylaminopropyl) carbodiimide hydrochloride and 0.05 M N-hydroxysuccinimide at a flow rate of 10 μL/min to create a reactive succinimide ester surface. Recombinant human CD109 (R&D systems) (20 μg/mL, prepared in 10 mM sodium acetate buffer, pH 5.0) was then coupled to the sensor via free amine coupling to the immobilized succinimide, followed by quenching the remaining activated succinimide esters with 1 M ethanolamine, pH 8.5, to immobilize the ligand and block the chip. The association phase involved flowing a few μM level analytes over the ligand-fixed channels at a flow rate of 30 μL/min, and the dissociation phase was determined by flowing the running buffer at the same rate. To identify optimal regeneration conditions, 20 mM NaOH was injected as a regeneration solution after the association/dissociation steps at a flow rate of 30 μL/min. KSHV was prepared in PBS (0.01 M phosphate buffer, 2.7 mM KCl, 0.137 M NaCl, pH 7.4) as the running buffer and serially diluted to achieve more than 3 concentrations. The association and dissociation times were set at 3 minutes each.

### Virion neutralization assay.

For the neutralization of KSHV by CD109 protein, the KSHV stock was mixed with recombinant human CD109 (4385-CD-050; R&D Systems) in PBS and incubated at 4°C for 1 hour with horizontal rotation to ensure thorough mixing. Following the incubation, HuARLT cells were infected with the virus-CD109 mixtures. At 24 hours of postinfection, the cells were detached using trypsin-EDTA and the infectivity was analyzed by measuring GFP expression using a Guava Flow Cytometer (Luminex).

### Immunohistochemistry.

Immunohistochemical staining was performed as previously described (62, 65) using rabbit polyclonal anti-CD109 antibody (1:100 dilution; 486955; Cell Signaling Technology) and alkaline phosphatase-conjugated goat anti-rabbit IgG (1:50 dilution; A3687; Sigma-Aldrich). Sections were developed with Vector Red AP substrate (Vector Laboratories) and counterstained with hematoxylin. 

### Statistics.

All experiments shown in this paper were performed at least 2 times independently, and representative data are shown. In graphs, results are presented as the means ± SD. To compare 2 different groups, 2-tailed Student’s *t* test was used. For multiple comparisons to a single control group, Dunnett’s test was performed with Bonferroni’s correction to adjust for multiple testing. All data were considered significant at a *P* value of < 0.05.

### Study approval.

Human tissue samples were obtained with written informed consent from all participants under protocols approved by the Institutional Review Board of Eulji University Hospital (no. EMC 2021-06-007) in accordance with the Declaration of Helsinki.

### Data availability.

Values for all data points in graphs are reported in the Supporting Data Values file. The mass spectrometry proteomics data have been deposited in the ProteomeXchange Consortium (http://proteomecentral.proteomexchange.org) via the PRIDE partner repository (http://www.ebi.ac.uk/pride) with the dataset identifier PXD048212 (http://www.ebi.ac.uk/pride/archive/projects/PXD048212). Additional detailed information is available from the corresponding author on request.

## Author contributions

MJL and MSL designed the study. MJL, JHY, JL, YHK, BSP, JP, SHY, and CP conducted experiments. MJL, JHY, YHK, SMY, and MSL analyzed the data. MJL, YHK, and JHY wrote the manuscript. MJL and JHY contributed to the experiments for the revision. DW, SJG, and MSL reviewed and edited the manuscript. SJG and MSL supervised the study. Co–first authors are listed in alphabetical order by last name.

## Supplementary Material

Supplemental data

Unedited blot and gel images

Supplemental table 1

Supporting data values

## Figures and Tables

**Table 2:**
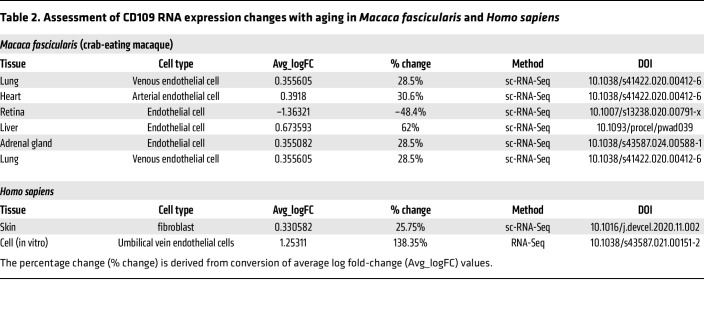
Assessment of CD109 RNA expression changes with aging in *Macaca fascicularis* and *Homo sapiens*

**Table 1:**
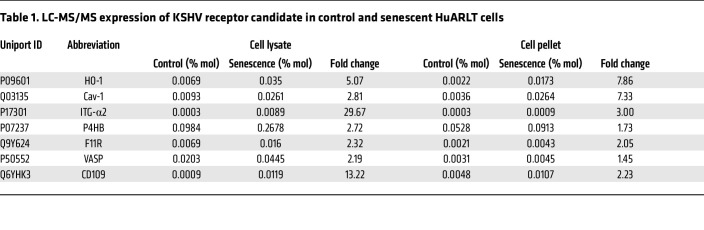
LC-MS/MS expression of KSHV receptor candidate in control and senescent HuARLT cells

## Version 1 12/12/2024

In-Press Preview

## Version 2 02/17/2025

Electronic publication

## Version 3 08/15/2025

Shou-Jiang Gao’s name corrected in author list.
