# Supplementary material for: Senescence of endothelial cells increases susceptibility to Kaposi’s sarcoma–associated herpesvirus infection via CD109-mediated viral entry
Source: J Clin Invest. 2024 Dec 12;135(4):e183561. doi: 10.1172/JCI183561 (PMC11827841; doi:10.1172/JCI183561)
Supplement: Supplemental data [file jci-135-183561-s012.pdf]

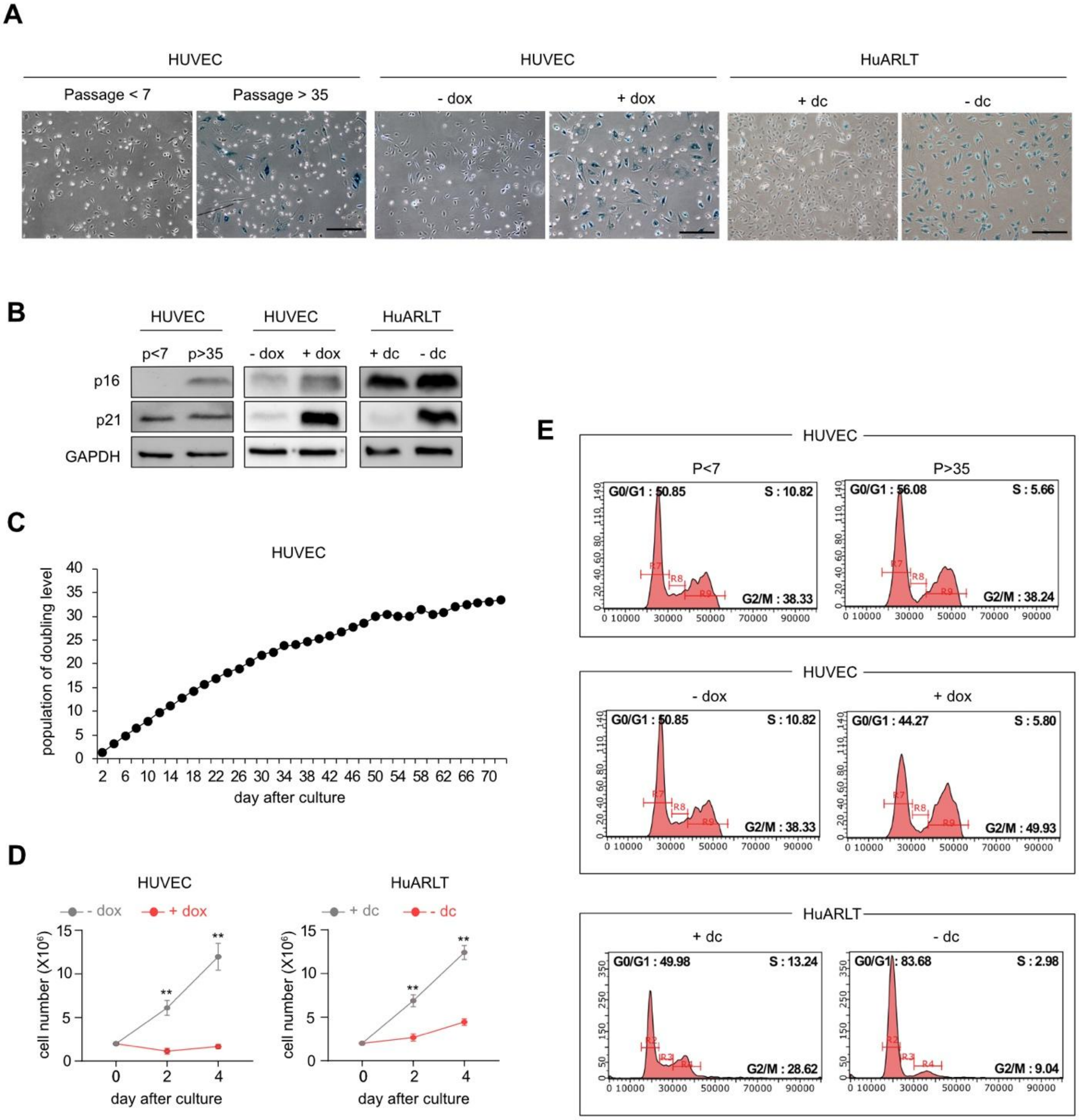

Supplemental Figure 1. Assessment of senescence of human endothelial cells. (A) SA-b-gal staining of senescent endothelial cells. Scale bar, 250 μm. **(B)** Western blot analysis for p16 and p21 in senescent endothelial cells. **(C)** Measurement of population of doubling level (PDL) in replicative senescent HUVECs. **(D)** Cumulative cell proliferation of HUVECs with doxorubicin and HuARLT cells without doxycycline. Data shown as mean ± SD, N = 3, **p < 0.01. **(E)** Cell cycle analysis for the non-senescent and senescent endothelial cells.

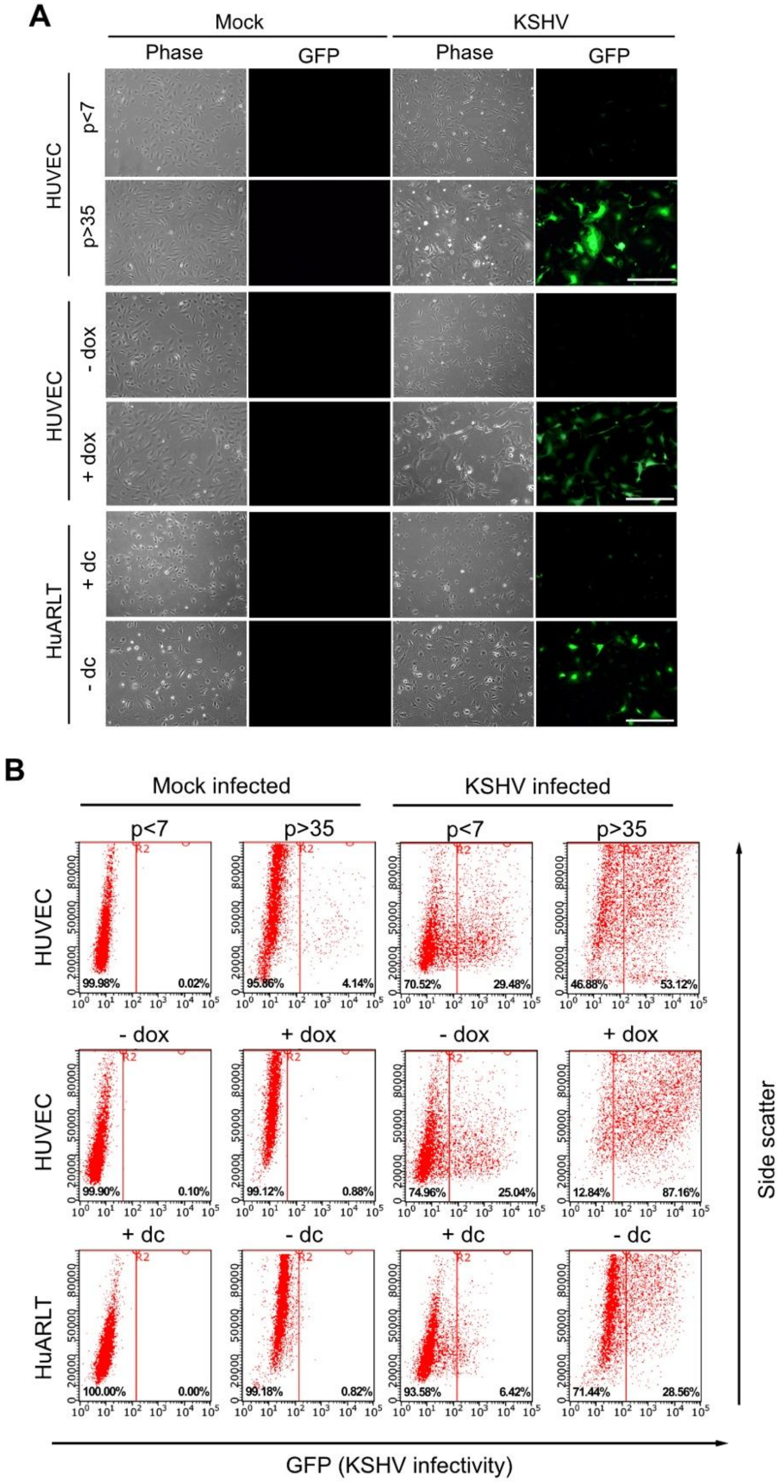

Supplemental Figure 2. KSHV infectivity in senescent human endothelial cells. (A) Fluorescence microscopy of KSHV-infected non-senescent and senescent human endothelial cells. KSHV infectivity was measured by GFP expression in cells infected with recombinant KSHV BAC16. Scale bar, 250 μm. **(B)** Representative analysis of flow cytometry for KSHV-infected cells.

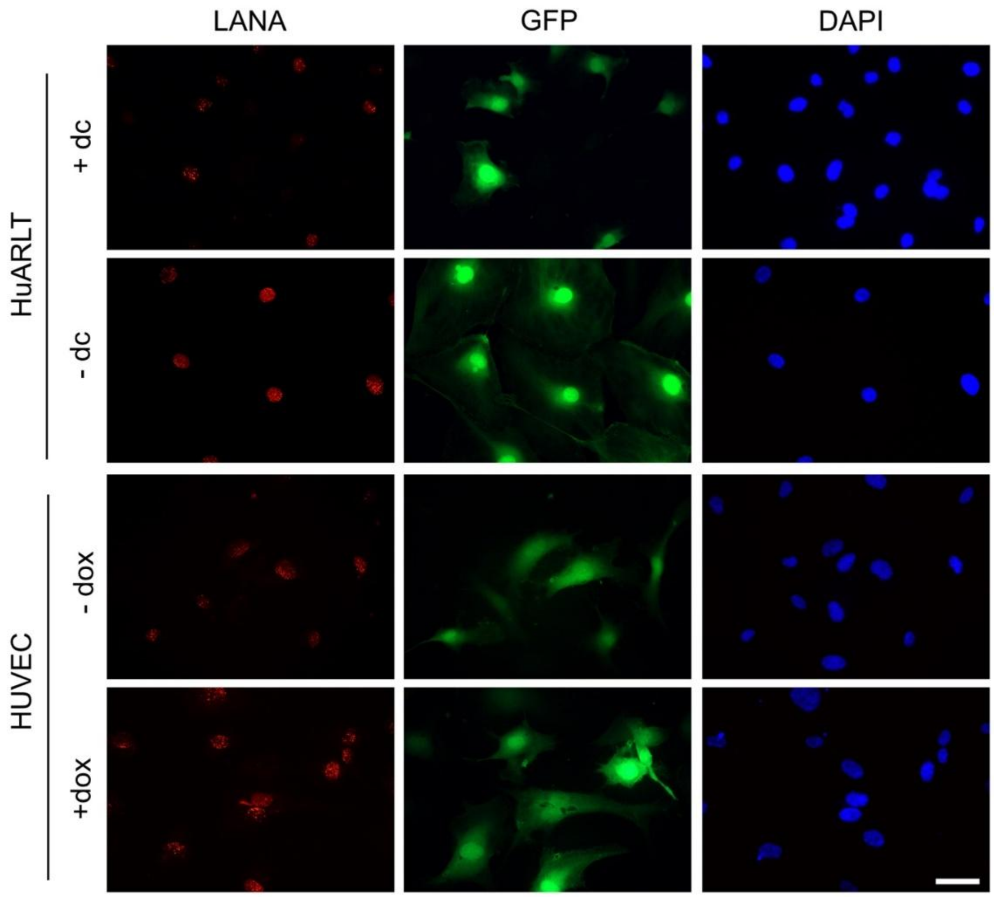

Supplemental Figure 3. Immunofluorescence assay for KSHV LANA in KSHV-infected cells in control and senescent human endothelial cells. DAPI was used to stain the nucleus of cells. Cells were fixed with 4% paraformaldehyde and permeabilized with 0.25% Triton X-100 (15 min). KSHV LANA was detected using rat monoclonal anti-HHV8 antibody (overnight, 4°C) followed by Alexa Fluor 568-conjugated goat anti-rat antibody (15 min, 4°C). Nuclei were counterstained with DAPI. Nikon Eclipse E400 microscope equipped with a Digital Sight DS-U2 camera and analyzed with NIS-Elements software. Scale bar, 50 µm.

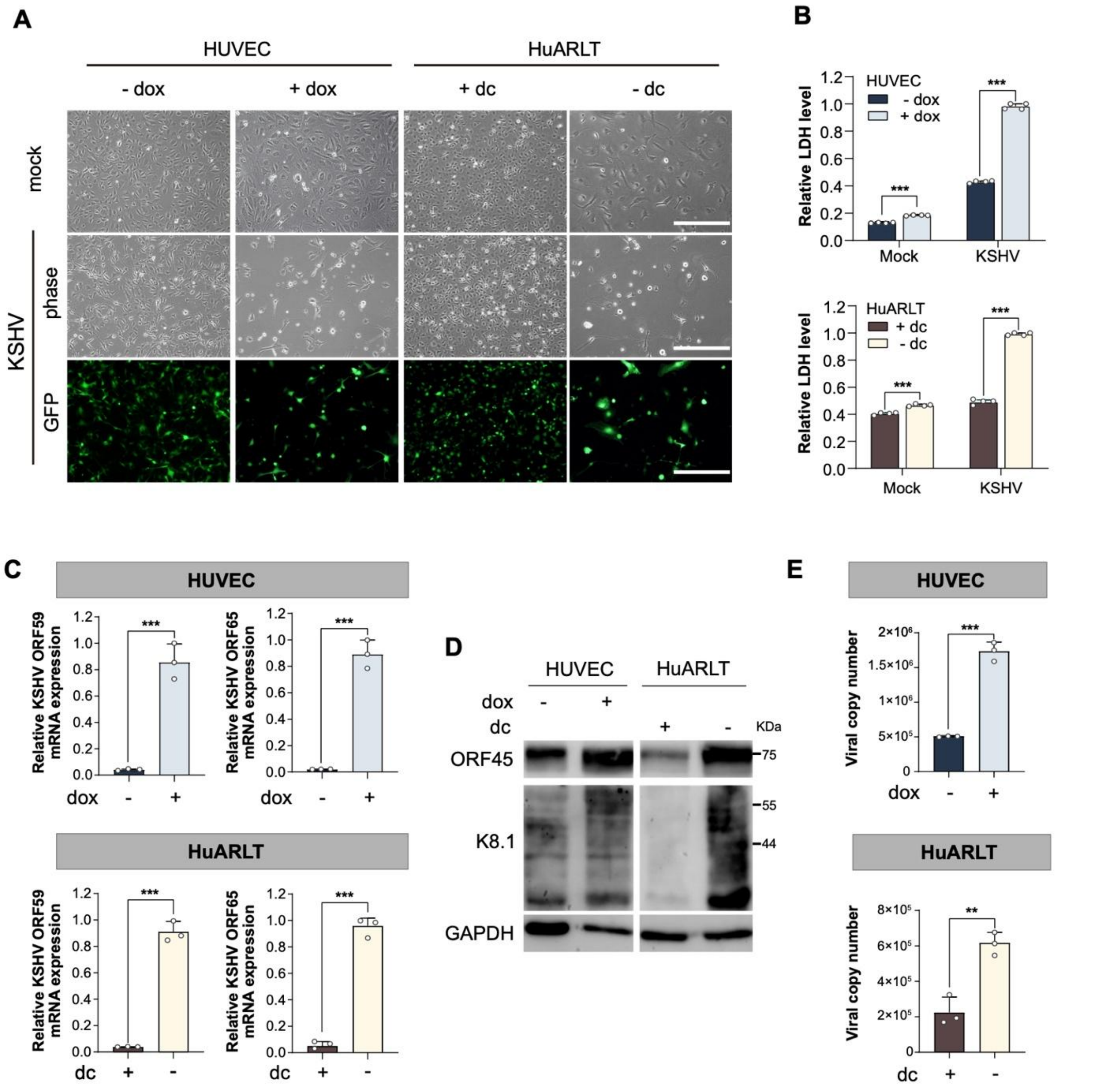

Supplemental Figure 4. Increased lytic replication in senescent endothelial cells after KSHV infection. (A) Microscopic cellular images of non-senescent and senescent endothelial cells following four days of infection with KSHV. Scale bar: 250 μm. **(B)** Lactate dehydrogenase (LDH) assay in non-senescent and senescent endothelial cells following four days of infection with KSHV. Data are presented as mean ± SD; N = 4 biological replicates, ***p < 0.001. **(C)** Relative mRNA expression levels of KSHV lytic replication markers in non-senescent and senescent endothelial cells following four days of infection with KSHV. Data are presented as mean ± SD; N = 3 biological replicates, ***p < 0.001. **(D)** Western blot analysis of KSHV lytic protein expression in non-senescent and senescent endothelial cells following four days of infection with KSHV. **(E)** Quantification of viral gene copy numbers in the supernatants from non-senescent and senescent endothelial cells following four days of infection with KSHV. Data are shown as mean ± SD; N = 3, **p < 0.01 and ***p < 0.001.

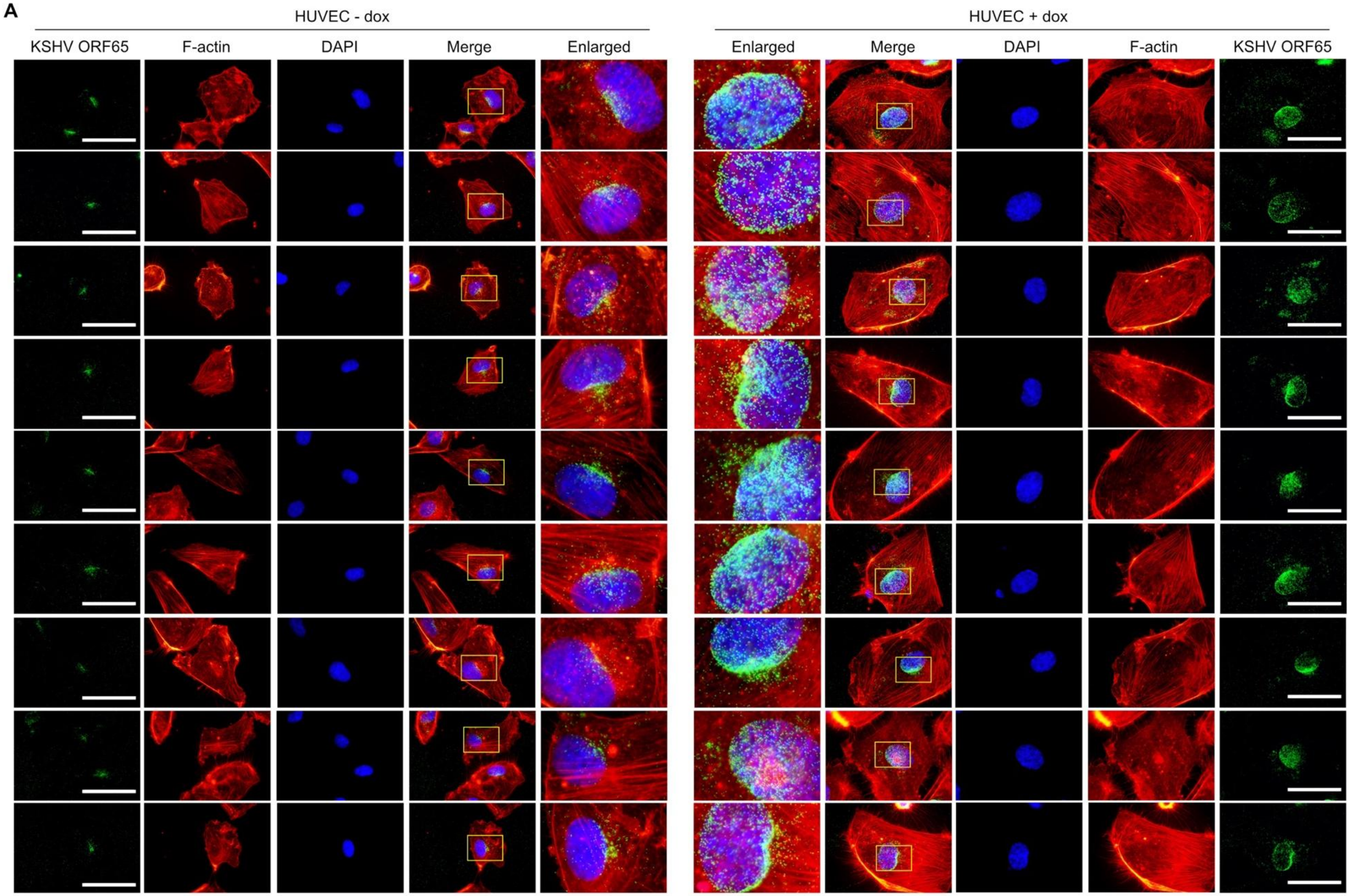
A
HUVEC - dox
KSHV ORF65
F-actin
DAPI
Merge
Enlarged
HUVEC + dox
Enlarged
Merge
DAPI
F-actin
KSHV ORF65

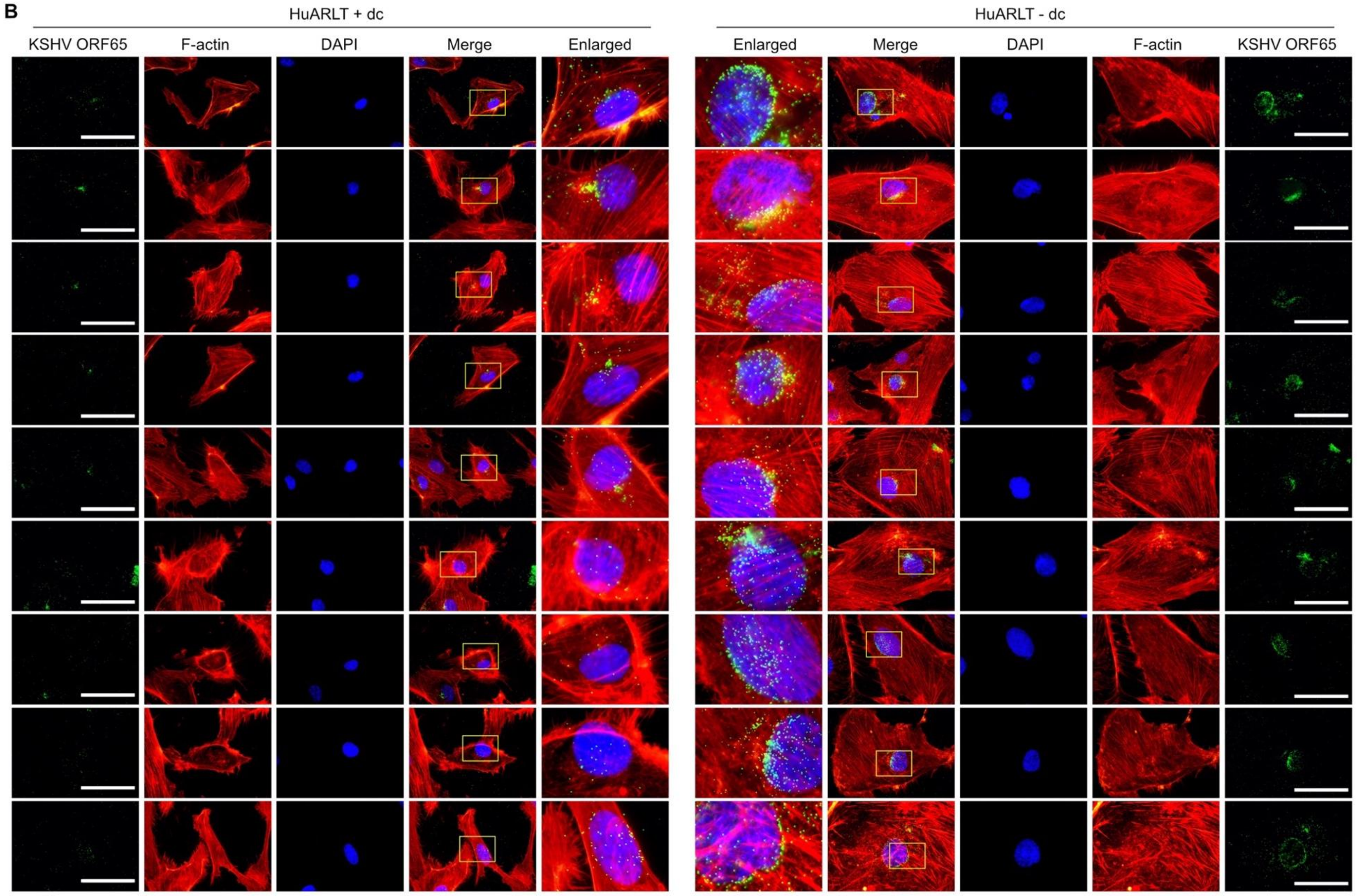

Supplemental Figure 5. Immunofluorescence assay for entry of KSHV in non-senescent and senescent cells of HUVEC (A) and HuARLT cells (B). Cells (1×10^5) were seeded on coverslips in 24-well plates and infected with equivalent volumes of KSHV the following day. After 4 hours of incubation

(37°C, 5% CO_2), cells were fixed with 4% paraformaldehyde and permeabilized with 0.25% Triton X-100 (15 min). KSHV was detected using mouse monoclonal anti-HHV-8 ORF65 antibody (overnight, 4°C) followed by Alexa Fluor 488-conjugated anti-mouse antibody (15 min, 4°C). F-actin was stained with Alexa Fluor 568 phalloidin, and nuclei were counterstained with DAPI. Images were captured using a ZEISS LSM 880 confocal microscope and analyzed with ZEISS Zen Blue Edition software. Viral particles were quantified using Nikon NIS software. Scale bar, 50 μm.

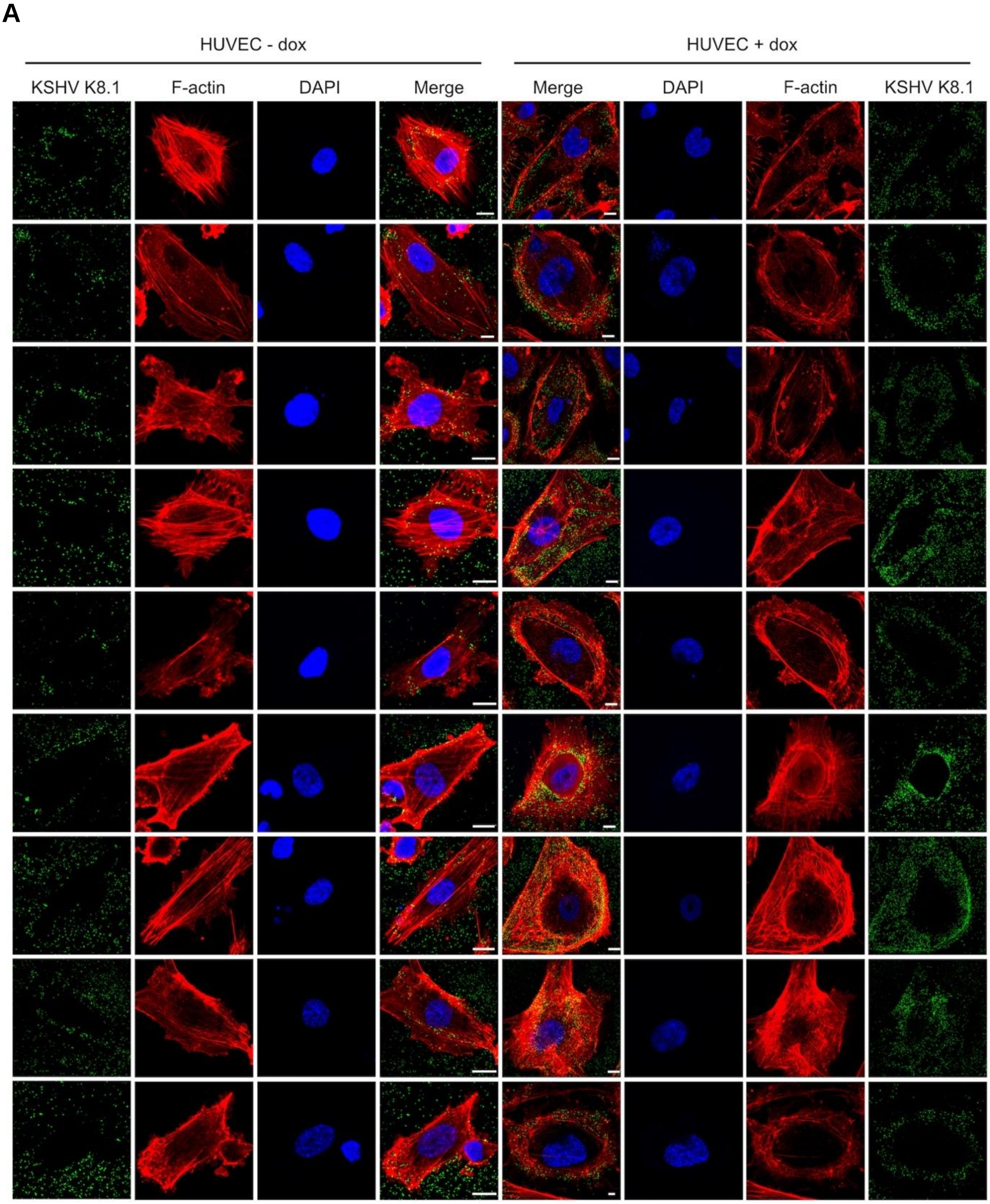
A
HUVEC - dox
HUVEC + dox
KSHV K8.1
F-actin
DAPI
Merge
Merge
DAPI
F-actin
KSHV K8.1

B

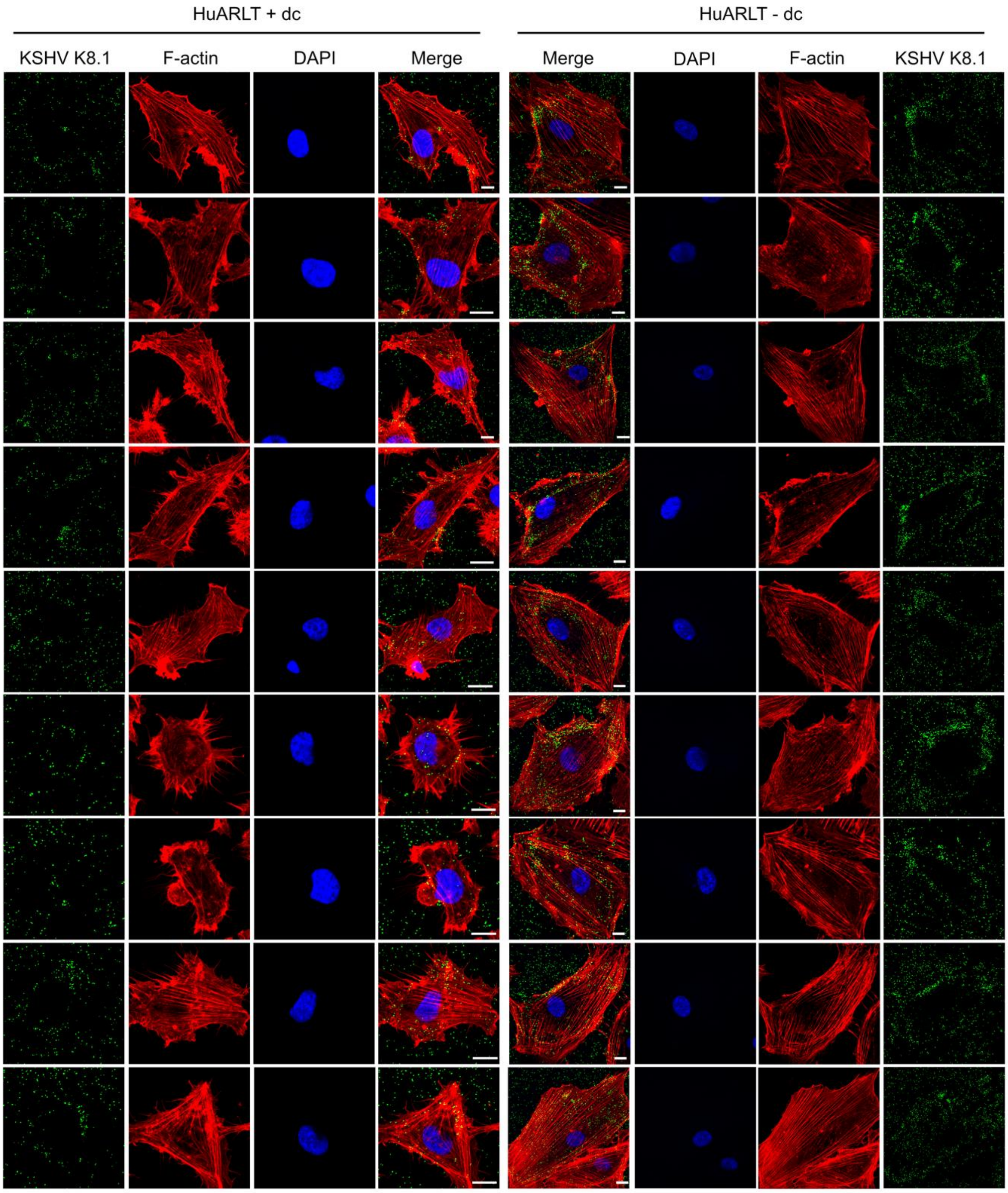

Supplemental Figure 6. Confocal microscopy images for KSHV binding to cell surface of HUVECs (A) and HuARLT cells (B). Cells (1×10^5) were seeded on coverslips in 24-well plates and infected with equivalent volumes of KSHV the following day. Post-infection, cells were washed with PBS and immediately fixed with 4% paraformaldehyde (30 min). Without permeabilization, KSHV was detected using mouse monoclonal anti-KSHV K8.1 antibody (overnight, 4°C) followed by Alexa Fluor 488-conjugated anti-mouse antibody (15 min, 4°C). F-actin was stained with Alexa Fluor 568 phalloidin, and nuclei were counterstained with DAPI. Images were captured using a ZEISS LSM 880 confocal microscope and analyzed with ZEISS Zen Blue Edition software. Viral particles were quantified using Nikon NIS software. Scale bar, 10 µm.

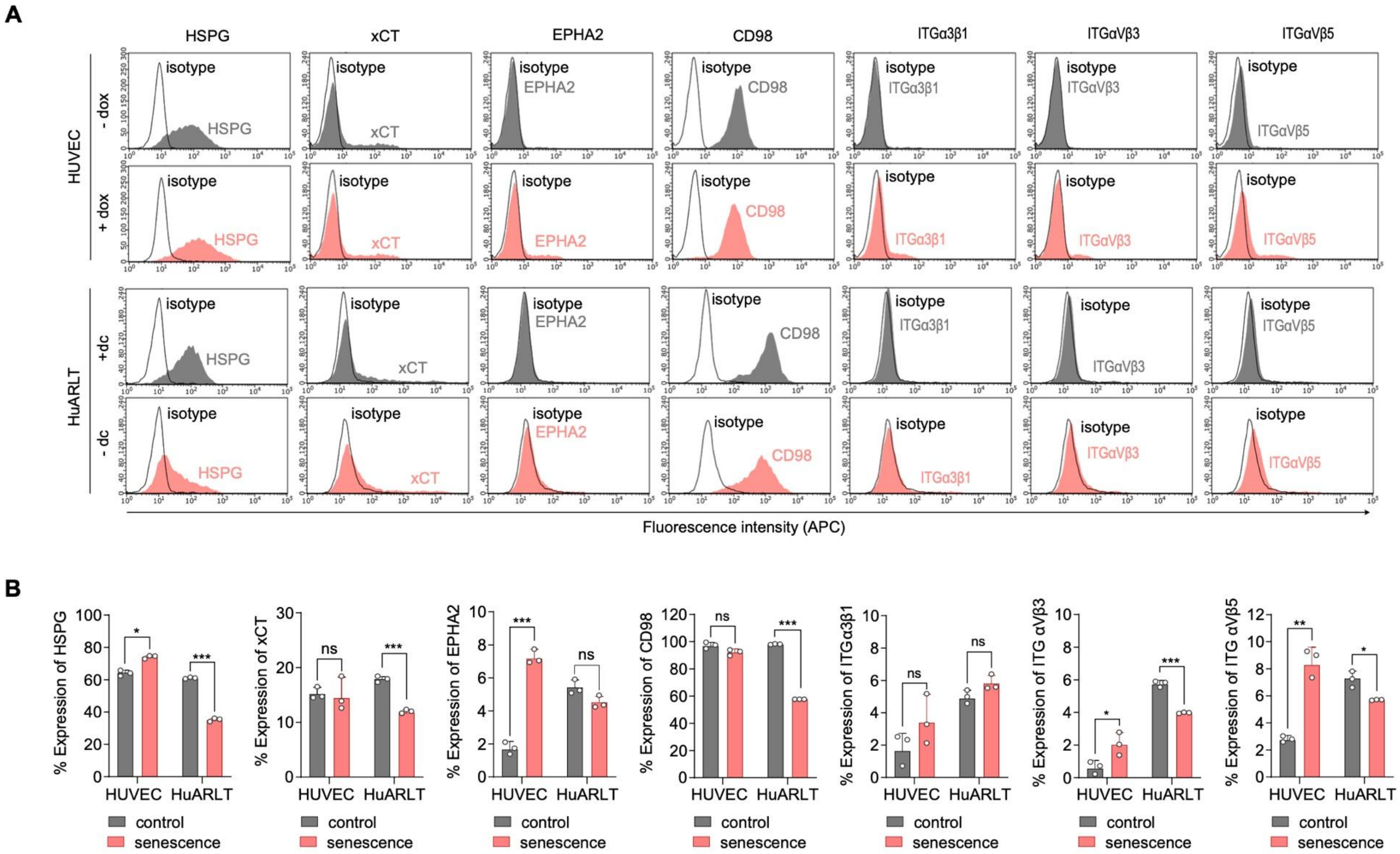

Supplemental Figure 7. Flow cytometry analysis of KSHV receptors expression in control and senescent endothelial cells using specific antibodies. HSPG, heparan sulfate proteoglycan; EPHA2, ephrin 2 type A receptor 2; ITG, integrin. (A) Representative results for each indicated receptor. **(B)** Analysis for receptor expressions on control or senescent endothelial cells. Data are presented as mean ± SD, N = 2, *p < 0.05, **p < 0.01, ***p<0.001, ns, not significant.

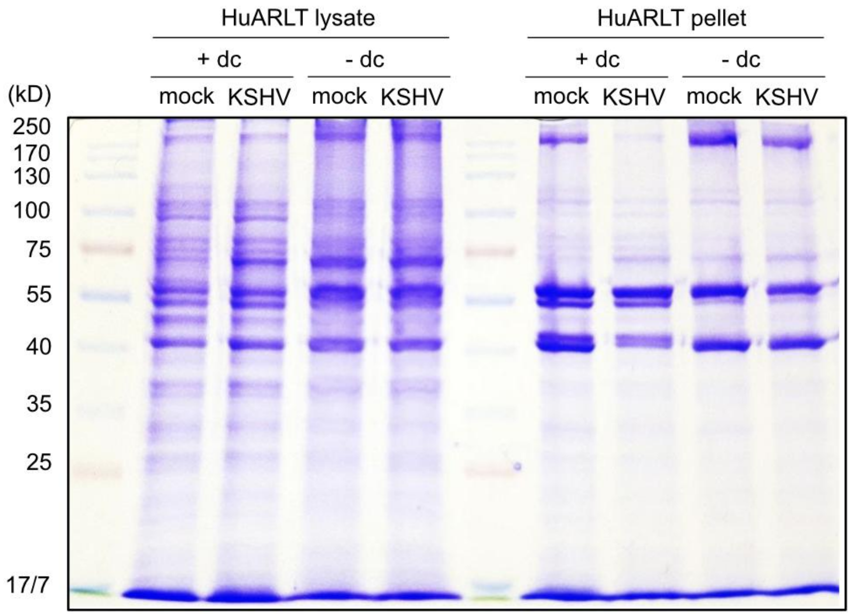

Supplemental Figure 8. Coomassie blue staining for SDS-PAGE gel with proteins from HuARLT cells. Control (+dc, culture with doxycycline) and senescent (-dc, culture without doxycycline) HuARLT cells were infected with KSHV or not (mock). The cell lysate was extracted using protein lysis buffer, and the cell pellet was collected from the undissolved pellet after obtaining cell lysate.

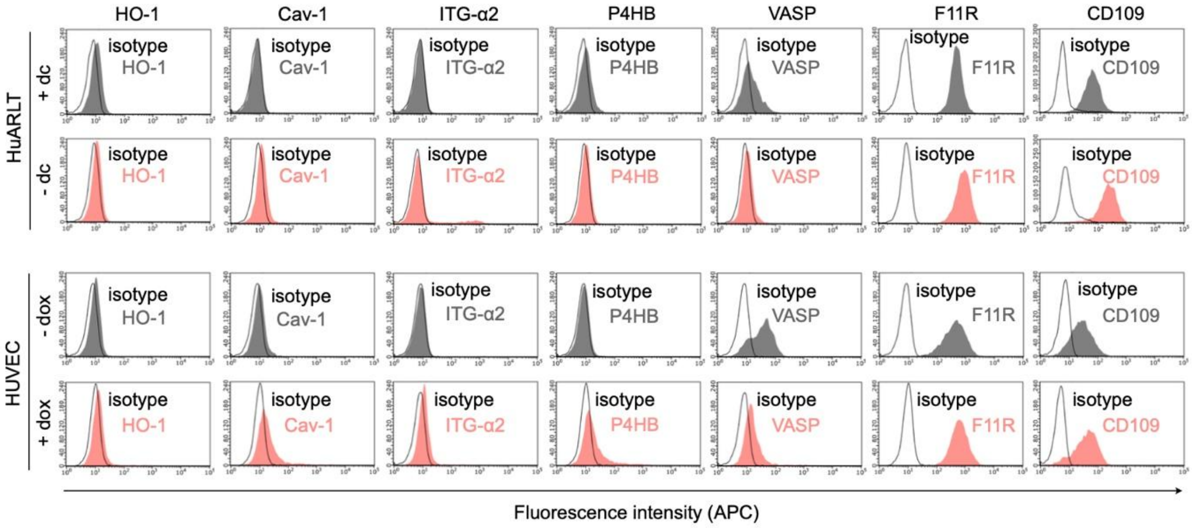

Supplemental Figure 9. Flow cytometric analysis of candidate proteins in non-senescent (+dc HuARLT and -dox HUVEC) and senescent (-dc HuARLT and +dox HUVEC) cells. Representative results and statistical analyses were presented in d and e, respectively.

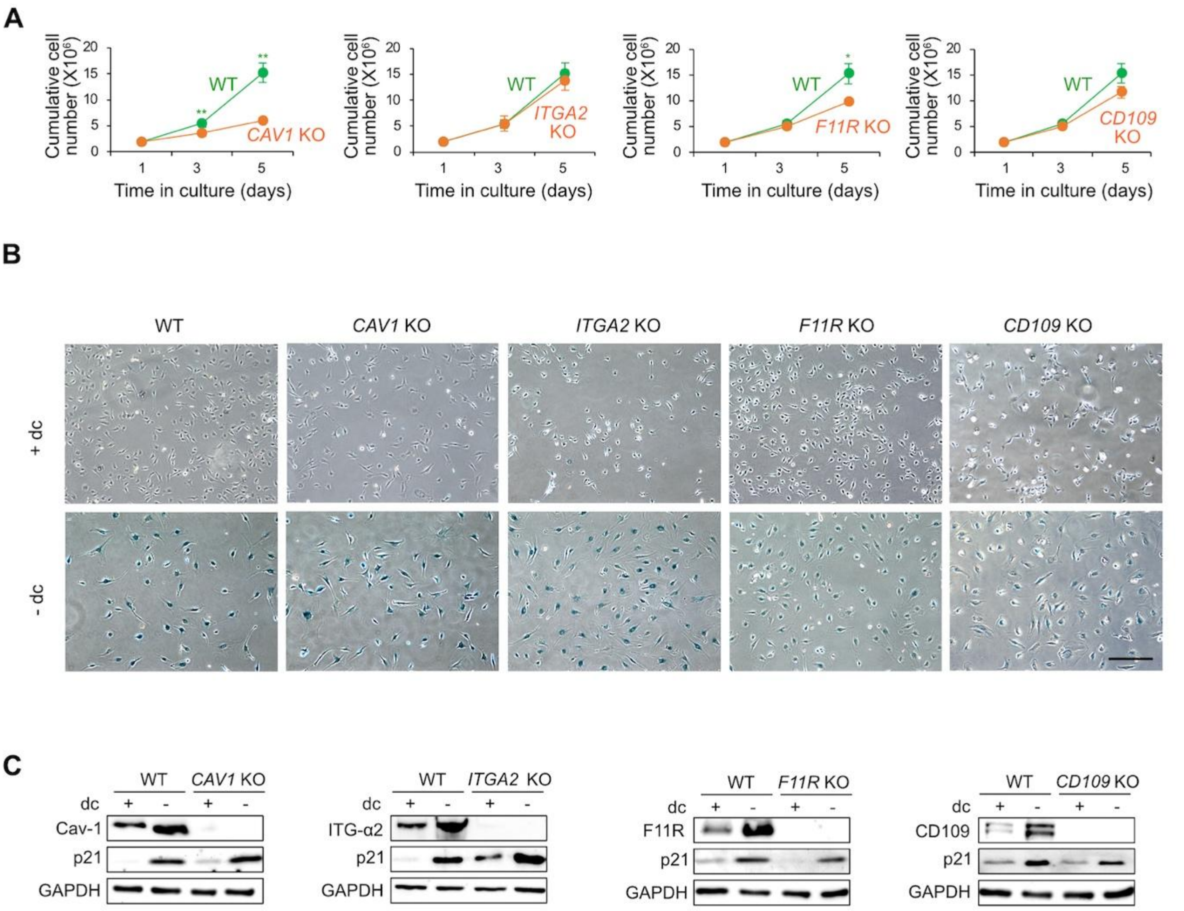

Supplemental Figure 10. Characterization of knockout clones from HuARLT cells. (A) The cumulative cell proliferation of WT and KO HuARLT cells. Viable cells from each group were counted every 2 days of subculture. Data shown as mean ± SD, N = 2; *p < 0.05, **p < 0.01. **(B)** SA-β-gal assay of control and senescent human endothelial cells for each KO clone. Scale bar, 250 µm. **(C)** Western blot analysis of p21 in each knockout clone. dc: doxycycline. GAPDH was used as a housekeeping protein for normalization.

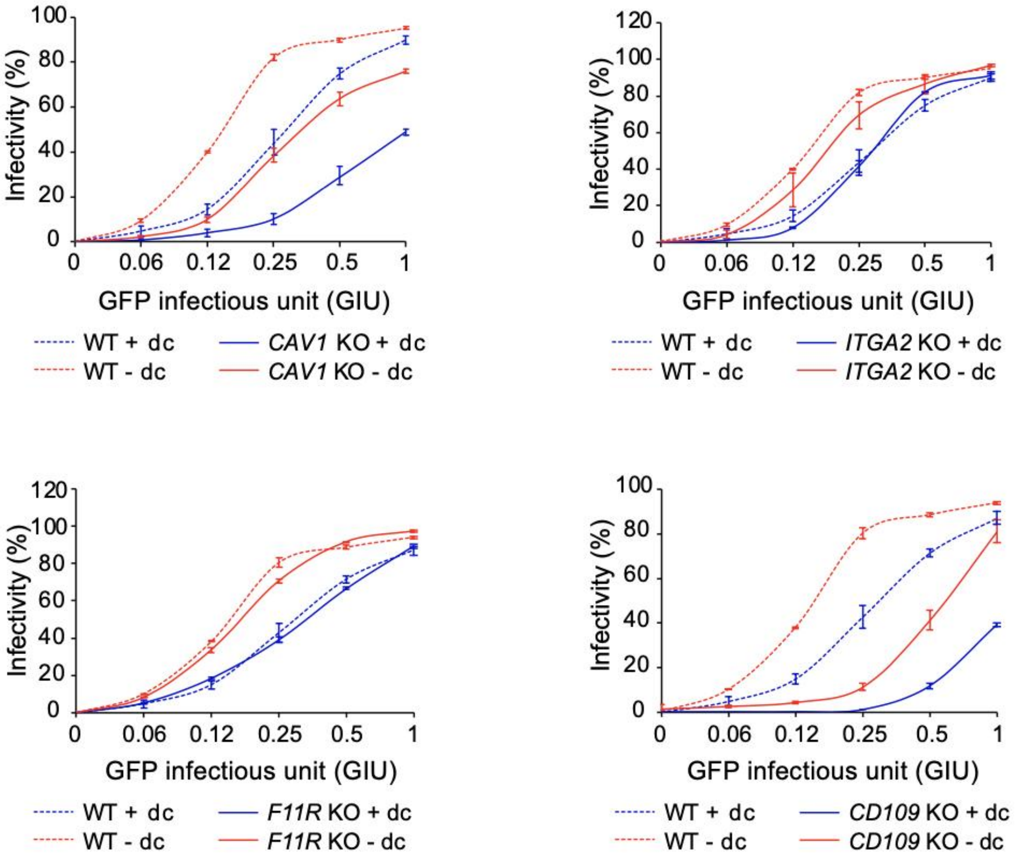

Supplemental Figure 11. Flow cytometric analysis of KSHV infectivity in knockout clones of *CAV1*, *ITGA2*, *F11R*, and *CD109*. An equivalent quantity of KSHV was infected in the same number of wild-type (WT) and knockout (KO) HuARLT cells, with or without induction of senescence. KSHV was prepared as a GFP infectious units of 1 to infect approximately 90% of non-senescent WT cells, followed by the infection of a 2-fold serially diluted virus into each conditioned cell. KSHV infectivity was measured using GFP expression. Flow cytometric analysis of KSHV-infected cells for each KO clone.

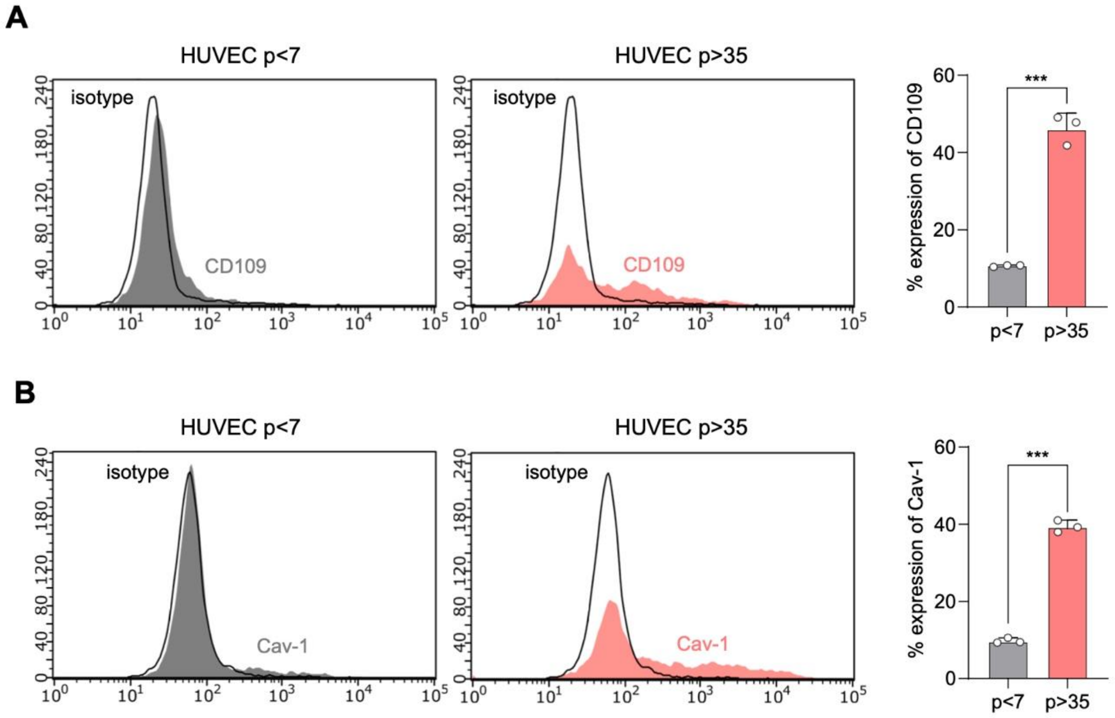

Supplemental Figure 12. Surface expressions of CD109 and caveolin-1. (A-B) Expression of CD109 (**A**) and caveolin-1 (cav-1, **B**) in control and senescent HUVECs through flow cytometry. Data shown as mean ± SD, N = 2, $*p < 0.05$, $**p < 0.01$.

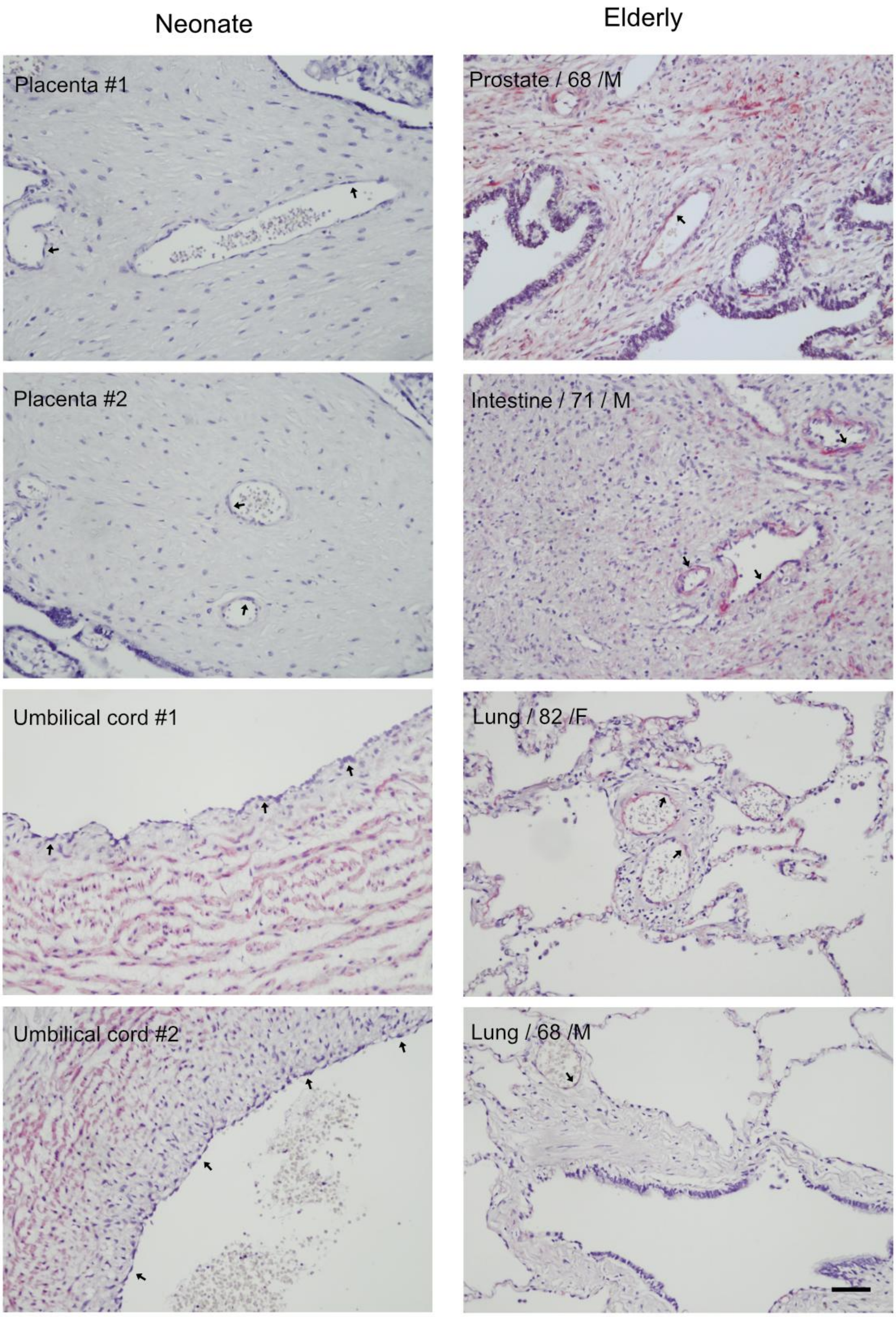

Supplemental Figure 13. Immunohistochemical detection of CD109 in tissues from neonates and elderly individuals. The left panel shows CD109 immunohistochemical staining of placenta (#1, #2) and umbilical cord (#1, #2) tissues from neonates. The right panel shows CD109 immunohistochemical staining of prostate (68-year-old male), intestine (71-year-old male), and lung tissues (82-year-old female and 68-year-old male). CD109 expression is visualized by red staining, and tissues were counterstained with hematoxylin. Black arrows indicate endothelial cells. Scale bar, 50 μm.

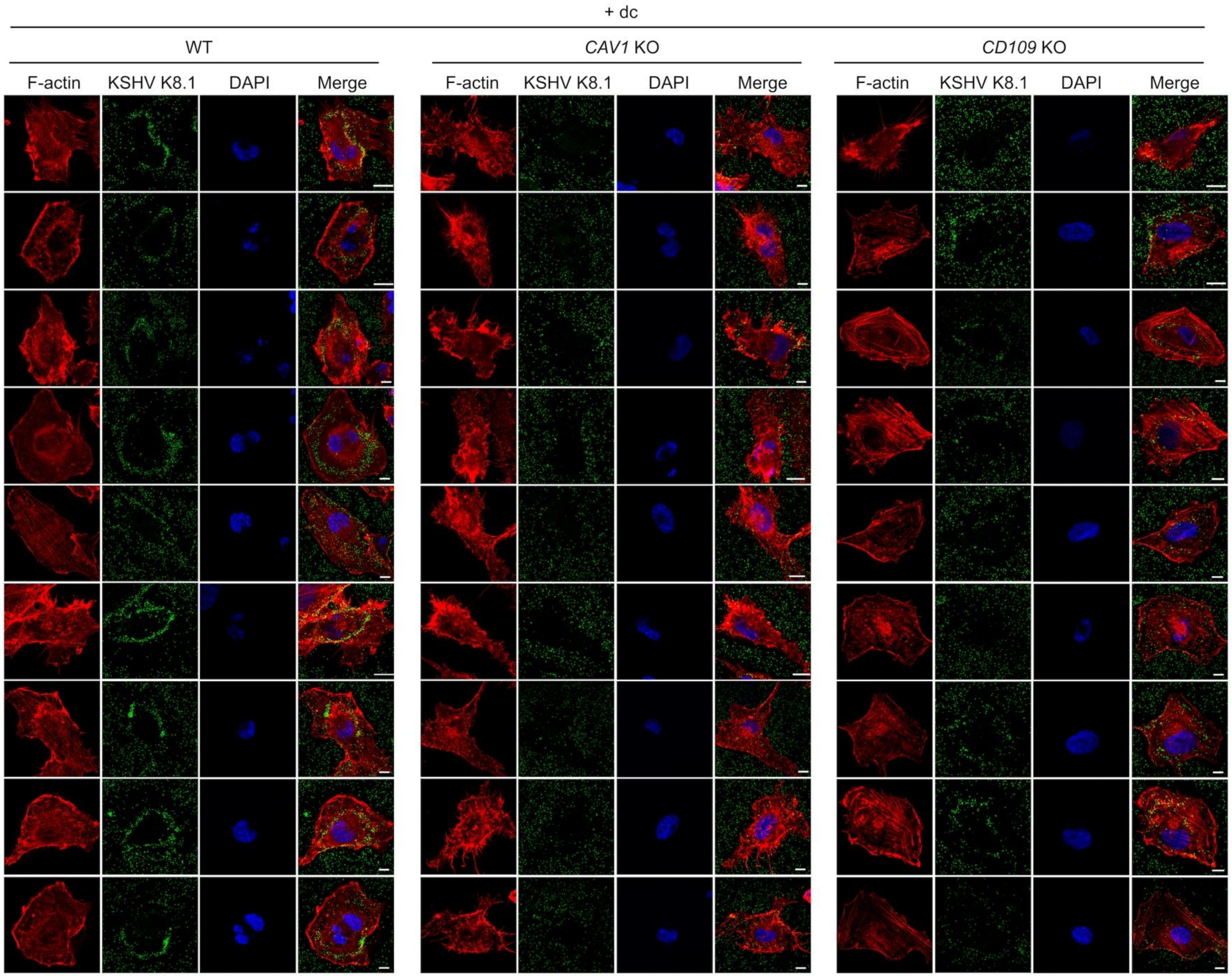

+ dc
WT
CAV1 KO
CD109 KO
F-actin
KSHV K8.1
DAPI
Merge

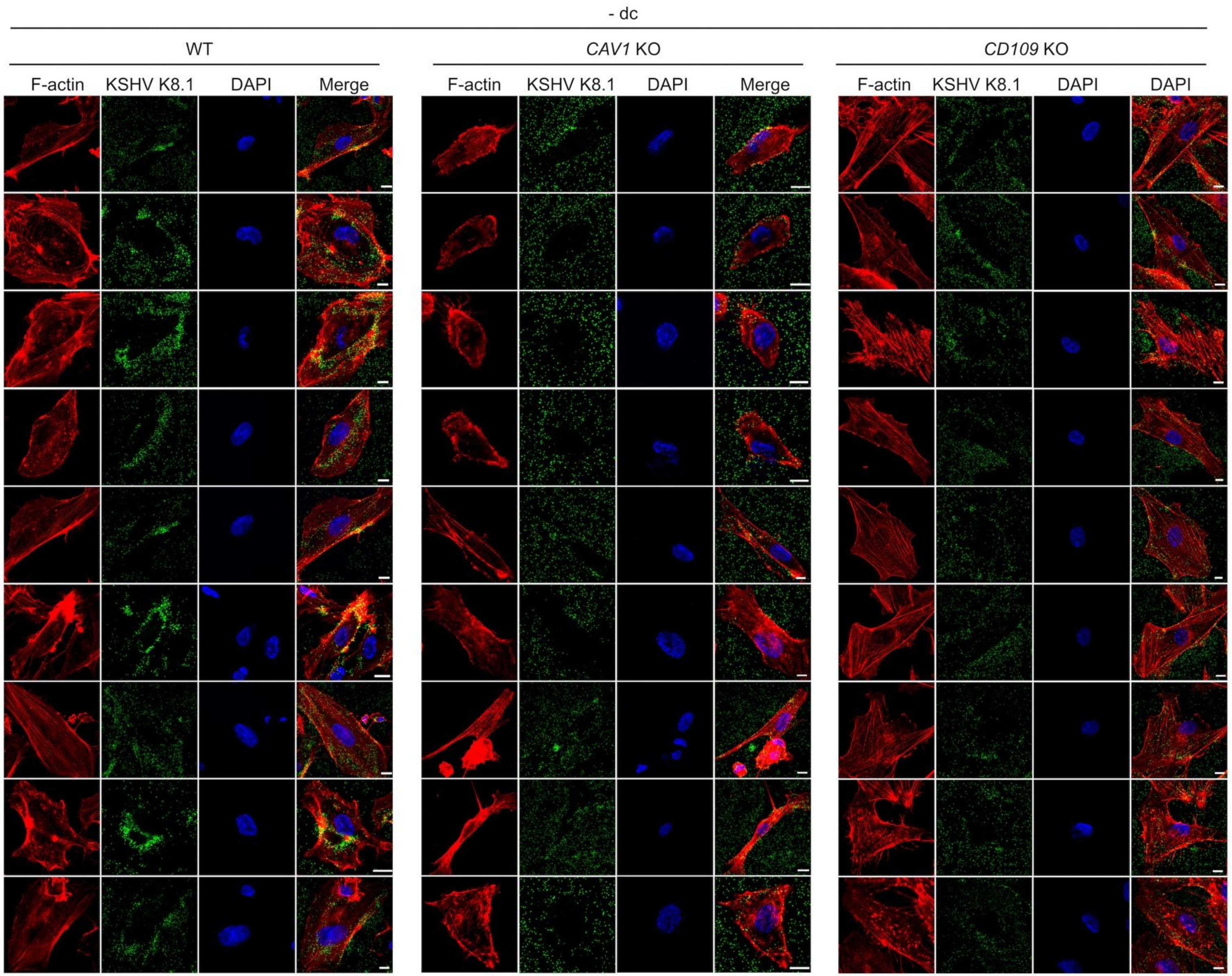

- dc
WT
CAV1 KO
CD109 KO
F-actin
KSHV K8.1
DAPI
Merge
F-actin
KSHV K8.1
DAPI
Merge
F-actin
KSHV K8.1
DAPI
DAPI

Supplemental Figure 14. Confocal microscopy images of KSHV virus particles in *CAV1* KO and *CD109* KO HuARLT cells. Cells (1×10^5) were seeded on coverslips in 24-well plates and infected with equivalent volumes of KSHV the following day. Post-infection, cells were washed with PBS and immediately fixed with 4% paraformaldehyde (30 min). Without permeabilization, KSHV was detected using mouse monoclonal anti-KSHV K8.1 antibody (overnight, 4°C) followed by Alexa Fluor 488-conjugated anti-mouse antibody (15 min, 4°C). F-actin was stained with Alexa Fluor 568 phalloidin, and nuclei were counterstained with DAPI. Images were captured using a ZEISS LSM 880 confocal microscope and analyzed with ZEISS Zen Blue Edition software. Viral particles were quantified using Nikon NIS software. Scale bar, 10 µm.

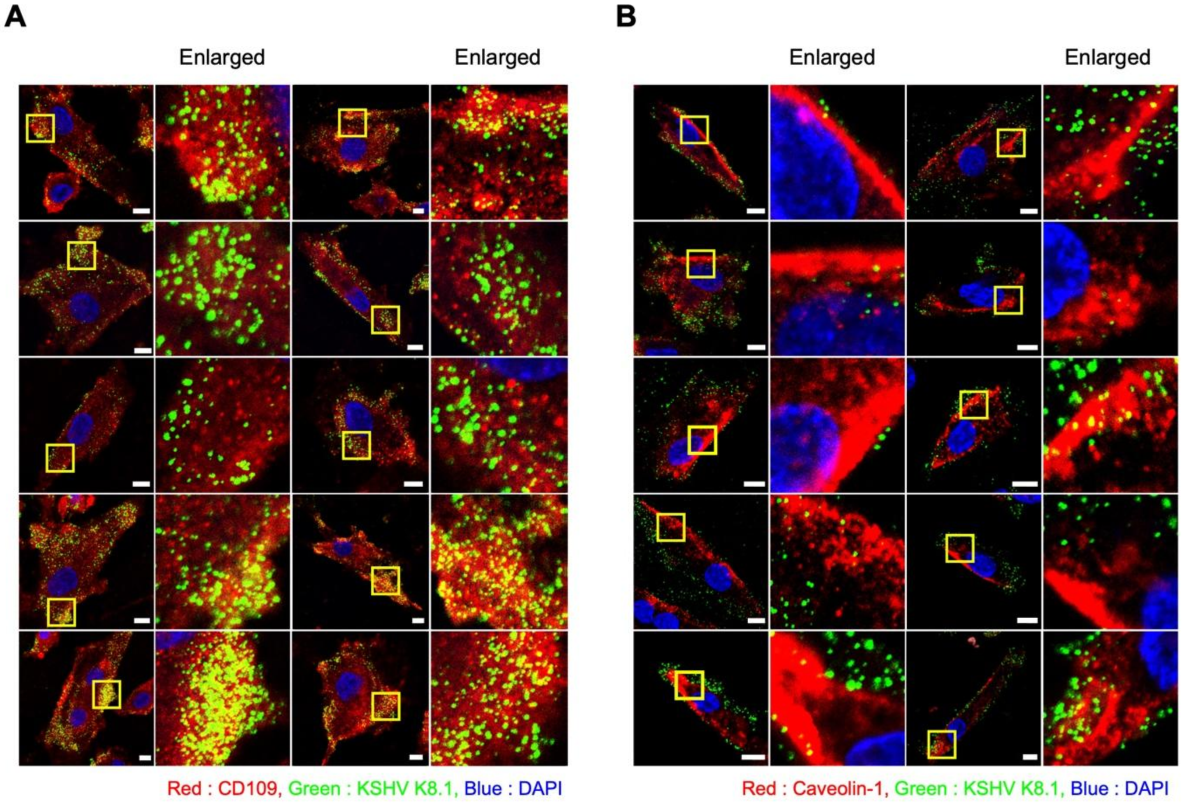

Supplemental Figure 15. Confocal microscopy images displaying colocalization of KSHV with CD109 (A) or caveolin-1 (B). Senescent HuARLT cells (1×10^5) were seeded on coverslips in 24-well plates and infected with equivalent volumes of KSHV the following day. Within 1 hour post-infection, cells were washed with PBS and fixed with 4% paraformaldehyde. Without permeabilization, KSHV was detected using mouse monoclonal anti-KSHV K8.1 antibody and Alexa Fluor 488-conjugated anti-mouse antibody. CD109 or caveolin-1 was stained with specific mouse antibodies and Alexa Fluor 568-conjugated anti-mouse antibody. Nuclei were counterstained with DAPI. Images were captured using a ZEISS LSM 880 confocal microscope and analyzed with ZEISS Zen Blue Edition software. Viral particles were quantified using Nikon NIS software. Scale bar, 10 μm.

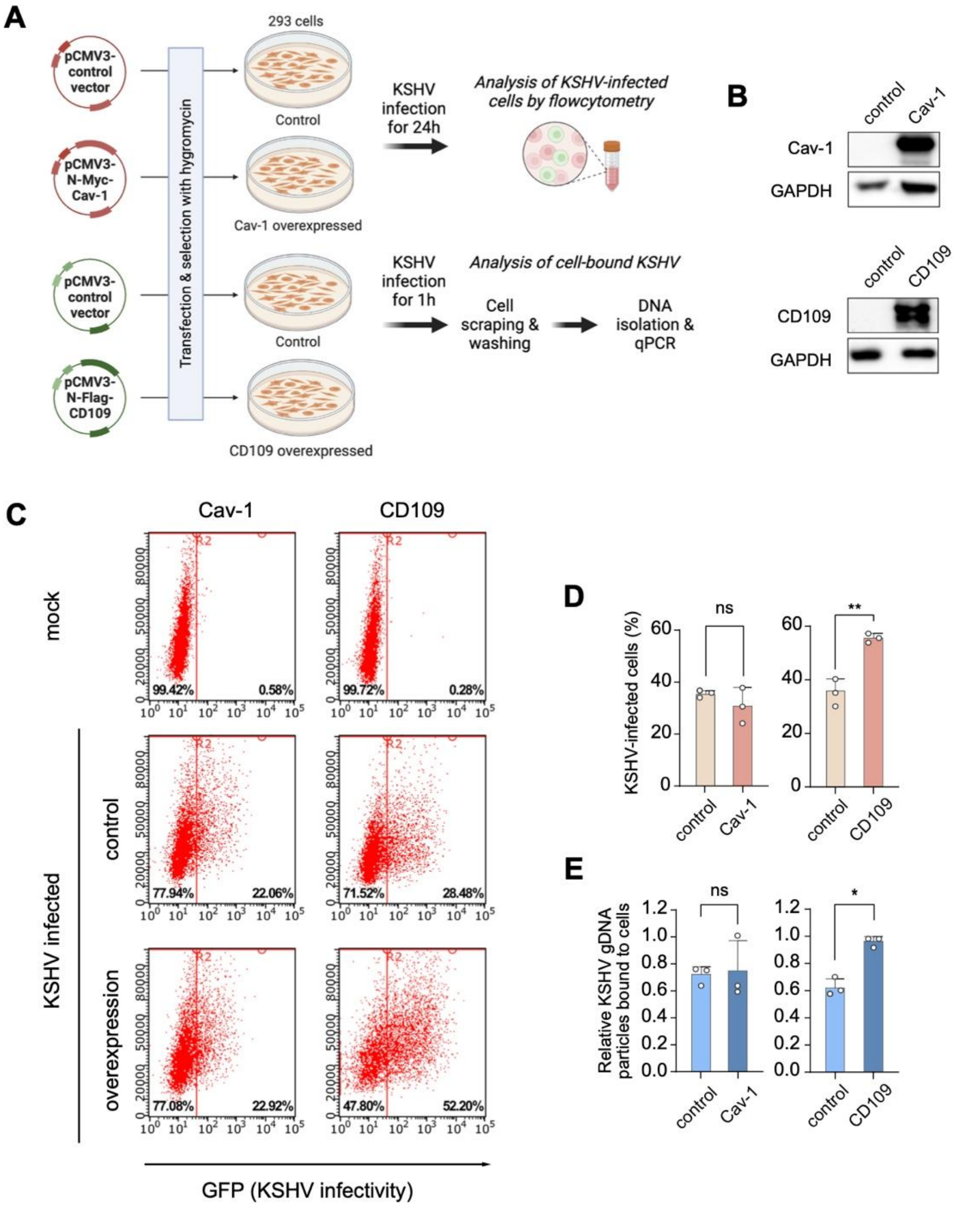

Supplemental Figure 16. Analysis of KSHV interaction with caveolin-1 and CD109 by overexpression of target genes. (A) Schematic representation of the establishment of caveolin-1 and CD109 overexpression and subsequent experimental approaches. Created by BioRender.com. **(B)** Western blot of target proteins caveolin-1 and CD109 overexpressed in 293T cells. The mock vector lacking the target gene served as a control. GAPDH was used for normalization. **(C-D)** Flow cytometric analysis of KSHV infectivity in cells overexpressing caveolin-1 or CD109. GFP expression in each cell was measured 1 day after infection. Representative images (**C**) and statistical analysis (**D**) of KSHV infectivity. Data are presented as mean ± SD, N = 3; $^{**}p < 0.01$, ns: not significant. **(E)** Quantification of KSHV binding to cells overexpressing the target gene by quantitative PCR. KSHV binding to the cells overexpressing the target gene was compared to control cells. The data were normalized to a housekeeping gene and is presented as mean ± SD, N = 3; $^{*}p < 0.05$, ns: not significant.
