# Supplementary material for: Senescence of endothelial cells increases susceptibility to Kaposi’s sarcoma–associated herpesvirus infection via CD109-mediated viral entry
Source: J Clin Invest. 2024 Dec 12;135(4):e183561. doi: 10.1172/JCI183561 (PMC11827841; doi:10.1172/JCI183561)
Supplement: Unedited blot and gel images [file jci-135-183561-s013.pdf]

Full unedited blot for Figure 4 B.

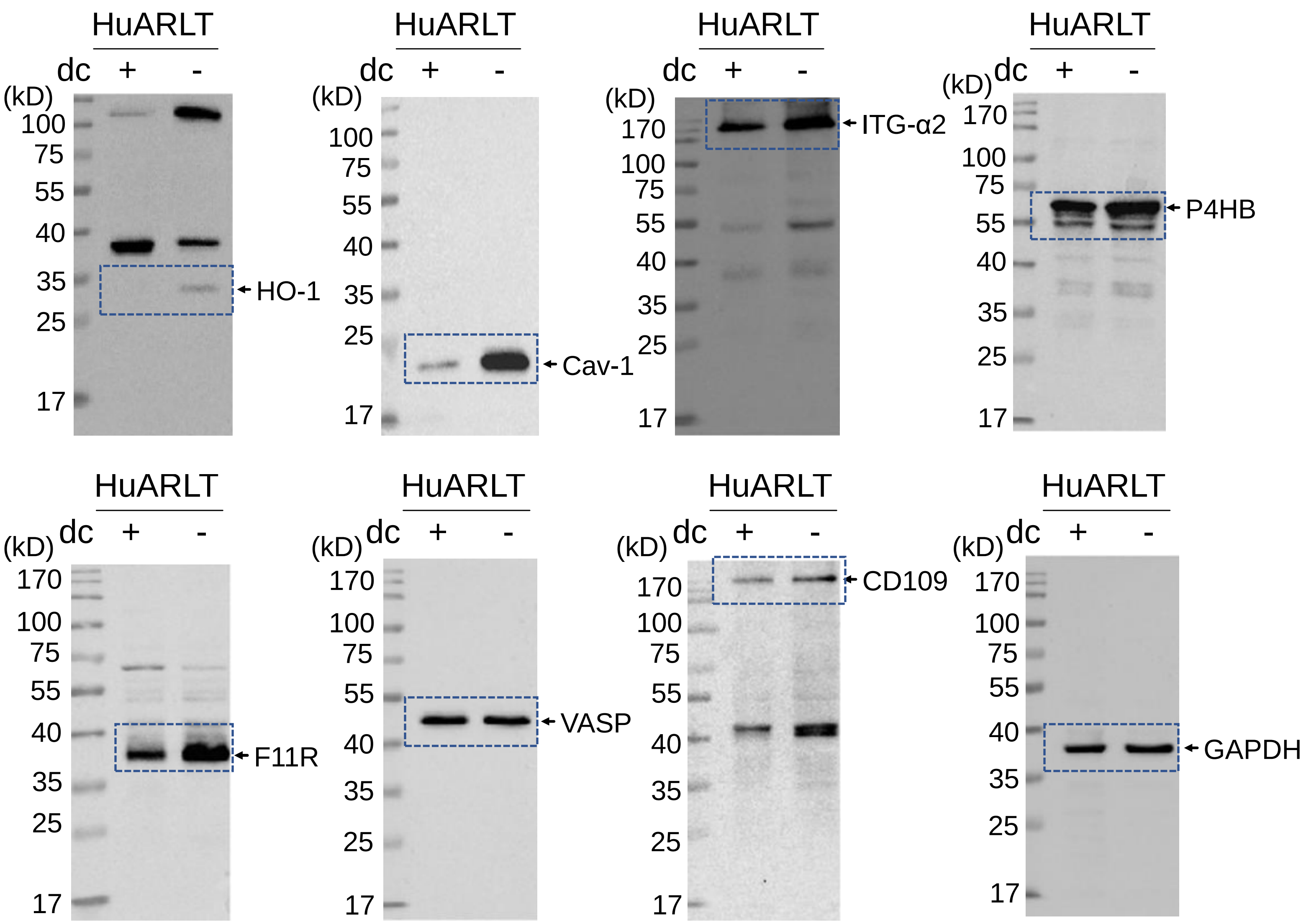

The square dotted boxes indicate the bands used in the figures.

Full unedited blot for Figure 5 B.

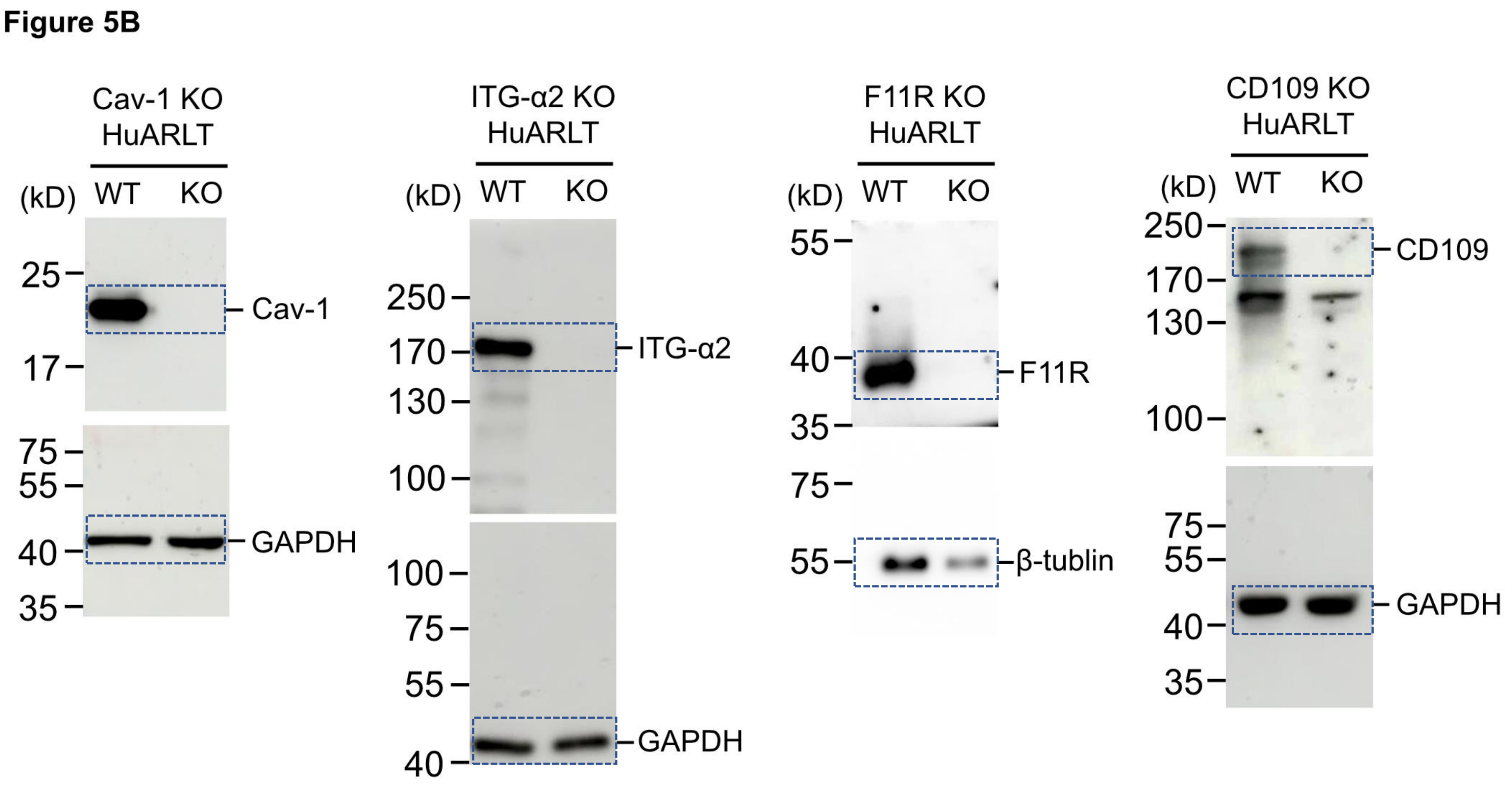

The square dotted boxes indicate the bands used in the figures.

Full unedited blot for Figure 9 A.

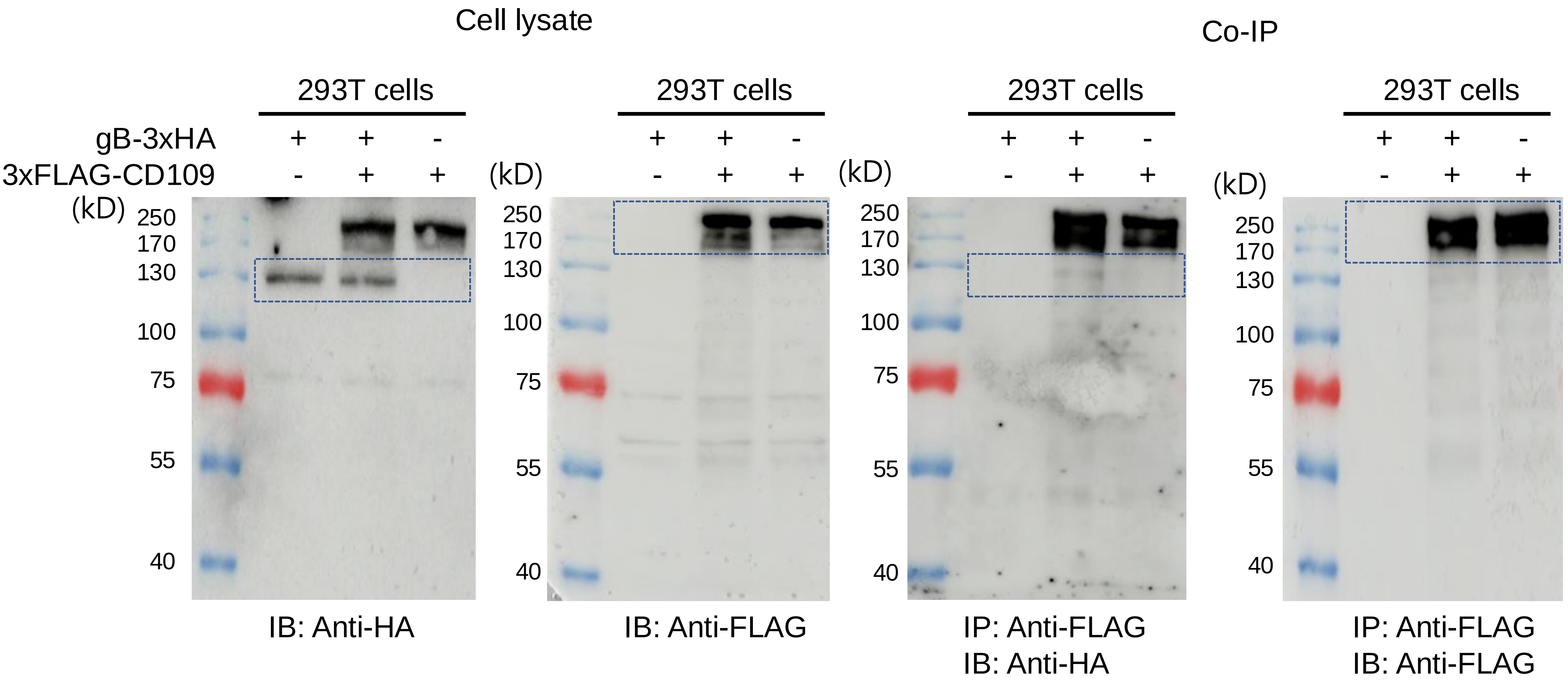

The square dotted boxes indicate the bands used in the figures.

Full unedited blot for Figure 9 B.

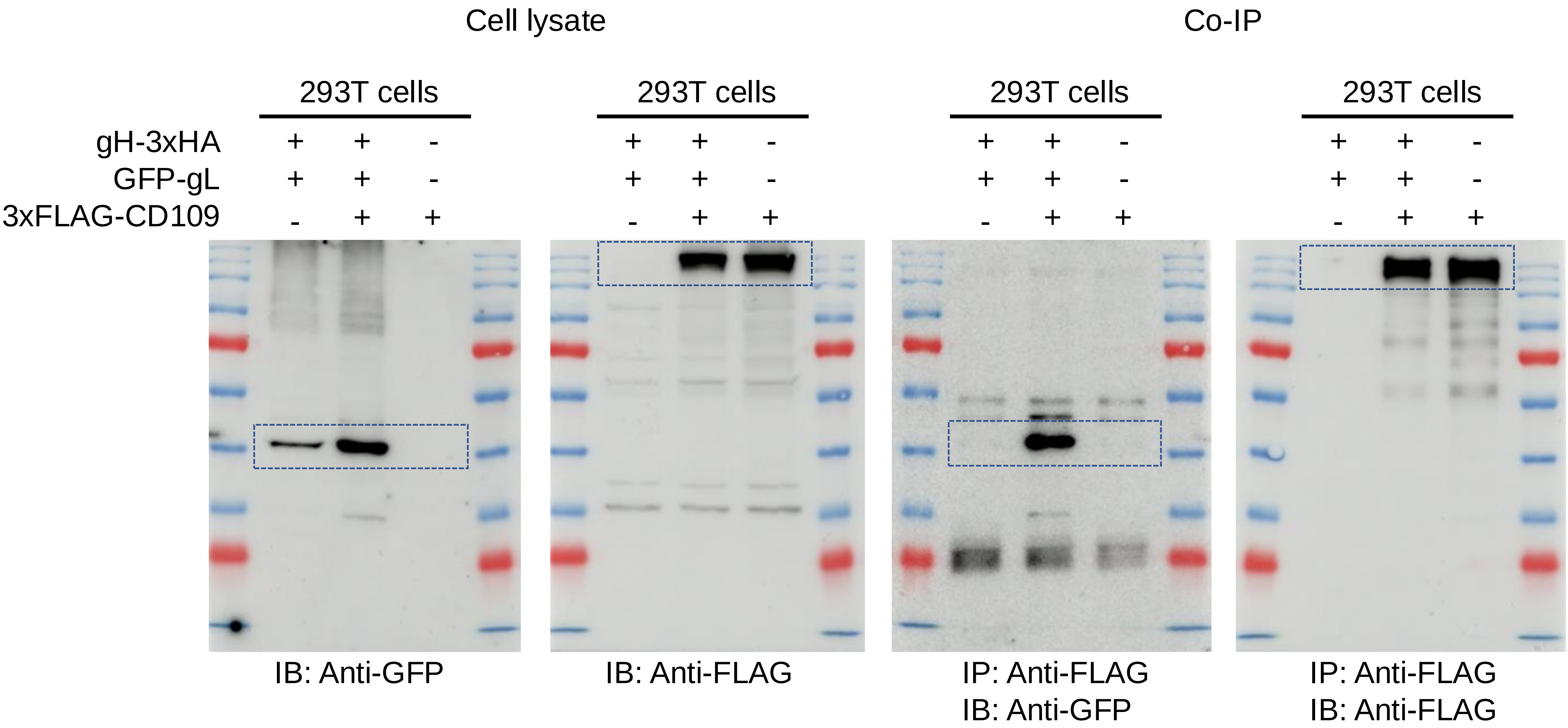

The square dotted boxes indicate the bands used in the figures.

Full unedited blot for Figure 9 C.

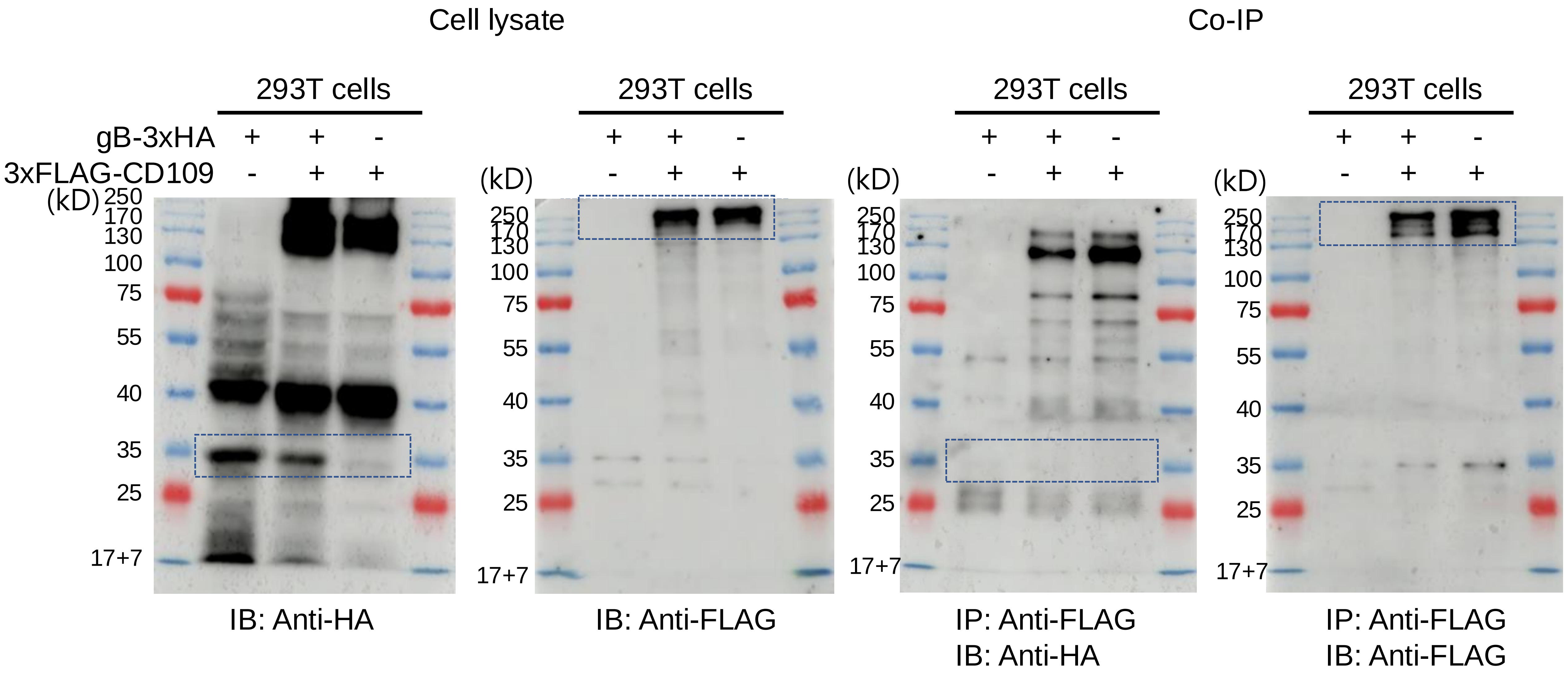

The square dotted boxes indicate the bands used in the figures.
